# Engineering of CD8^+^ T cells with an HIV-specific synthetic notch receptor to secrete broadly therapeutic antibodies for combining antiviral humoral and cellular immune responses

**DOI:** 10.1128/mbio.03839-24

**Published:** 2025-02-25

**Authors:** Lina Meng, Haichi Zhao, Shangkun Chang, Weiting Li, Yinghui Tian, Ruihong Wang, Libian Wang, Tiejun Gu, Jiaxin Wu, Bin Yu, Chu Wang, Xianghui Yu

**Affiliations:** 1 National Engineering Laboratory for AIDS Vaccine, School of Life Sciences, Jilin University, Changchun, China; 2 Key Laboratory for Molecular Enzymology and Engineering, the Ministry of Education, School of Life Sciences, Jilin University, Changchun, China; University of California, Davis, Davis, California, USA

**Keywords:** HIV-1, synNotch, broadly neutralizing antibody, T cell re-targeting antibody, CD8^+ ^T cell, cell engineering

## Abstract

The application of immunotherapeutic strategies, such as chimeric antigen receptor-T cells and broadly neutralizing antibodies (bNAbs), for the treatment of human immunodeficiency virus (HIV) infection is hindered by the latent reservoirs and viral escape. Achieving long-term control of viral load in the absence of antiretroviral therapy requires a combination approach utilizing these immunotherapeutic strategies. For this purpose, we developed novel anti-HIV-1 synthetic Notch (synNotch) receptor-T cells, termed CD4-17b-VN, which express both a bNAb (VRC01) and a bispecific T cell-engaging protein (N6-αCD3) with antigenic control. The synNotch receptor-expressing cells can sense the viral antigen presented on both HIV-1 particles and the surface of target cells. A human T cell line equipped with the CD4-17b-VN circuit could effectively control VRC01 and N6-αCD3 secretion upon sensitization, suppress the infection of diverse subtypes of HIV-1 strains, and mediate specific bypass cytotoxic activity against infected and latency-reactivated cells. Additionally, CD4-17b-VN CD8^+^ T cells exhibited long-lasting suppression of infected cells and stronger killing effect on latency-reactivated cells *in vitro*. Importantly, we demonstrated that the synNotch receptor did not increase susceptibility to HIV-1 infection in the engineered cells. Our study validates the concept of a synNotch platform-based T cell therapeutic approach that can deliver broadly therapeutic antibodies in an HIV-1 antigen-controlled manner, which may have important implications for the functional cure of AIDS.

**IMPORTANCE:**

Adoptive transfer of effector T cells modified with a chimeric antigen receptor has been proposed as an applicable approach to treat human immunodeficiency virus (HIV) infection. The synNotch receptor (SNR) system serves as a versatile tool, enabling customized programming of input and output functions in mammalian cells. Herein, we report a novel synNotch platform-based approach for T cell engineering targeting both cell-free particles and infected cells by coupling antibody neutralization with cytotoxicity. Our findings demonstrate that the engineered CD4-17b SNR enables controllable production of functional anti-HIV-1 broadly neutralizing antibody and bispecific T cell-engaging protein upon recognition of the viral particle and cell surface antigens by the bifunctional synNotch-T cells. Human primary CD8^+^ T cells equipped with the bifunctional synNotch circuit CD4-17b-VN can effectively suppress long-term viral replication and reduce latency-reactivated cells *in vitro*, without the undesired risk of being infected by the virus, suggesting their potential candidacy for AIDS therapy with prospects for future clinical applications.

## INTRODUCTION

Antiretroviral therapy (ART) can curb the replication of human immunodeficiency virus (HIV) to almost undetectable levels (1–3), but it cannot eradicate HIV infection due to the enormous barriers to viral reservoirs in infected cells (4, 5). Persistent viral infection and associated immune dysfunction accompanying long-term ART somewhat decrease the survival probability of people with HIV (6–9). These realities emphasize the need to develop strategies to reduce the viral reservoir and achieve sustainable control of viral rebound after ART withdrawal.

Previous studies have demonstrated that the HIV-specific cytotoxic T lymphocyte response is a key component of host immunity against HIV infection and is essential for viremia control and elimination of HIV-infected cells (10–12). Recent advances in harnessing T cells for acquired immunodeficiency syndrome (AIDS) therapy have led to innovative methods of targeting cytotoxic T cells to the viral envelope (Env) proteins on HIV-infected cells (13, 14). Anti-HIV-1 chimeric antigen receptor (CAR)-modified T cells, which typically use the single-chain fragment variables (scFvs) of anti-HIV-1 Env antibodies (14–17) or CD4 extracellular domains (18–21) as the recognition domains of CAR, have shown effective killing capacity against infected cells and the ability to reduce the reservoir size in animal models (18–23), providing a promising strategy to achieve a functional cure of AIDS in combination with latency-reversing agents (LRAs) (24, 25). However, despite some clinical trials demonstrating the long-term safety and durability of CAR-T cell infusions, they provide minimal clinical benefit for AIDS (13, 26–29). Limited cytolytic function *in vivo*, susceptibility to HIV infection, and poor proliferative capacity may have caused these disappointing results (30). To achieve durable virological control off-ART, strategies to enhance the functions of engineered T cells are necessary (24).

The ability of broadly neutralizing antibodies (bNAbs) to suppress viremia has also rendered it a noteworthy focus in AIDS therapy. Numerous high-affinity bNAbs targeting different epitopes on the Env trimer have been isolated from HIV-1-infected individuals, and their therapeutic efficacy has been evaluated in animal models (31–34) and clinical trials (35–39). bNAbs can neutralize cell-free viruses by directly blocking their binding to host cells and enhance HIV-1-specific immune responses through Fc-mediated activities. However, despite some advancements in bNAb therapies, which can maintain viral suppression for longer periods after ART cessation, the bNAbs alone are not able to be successful in curing AIDS because the virus rapidly escapes by selection of resistant variants (40).

Given the unlikely efficacy of a singular treatment modality in achieving a therapeutic cure (30, 41), there is an urgent need for novel strategies that combine bNAbs with cytotoxic T cells to effectively control viremia and eliminate HIV-infected cells. One approach involves linking the scFvs of bNAbs with those of antibodies that can bind to T cell antigens, typically the scFvs of anti-CD3 antibodies, to form bispecific molecules such as dual-affinity re-targeting proteins (DARTs) and bispecific T cell-engaging proteins (BiTEs) (42–44). These molecules directly recruit cytotoxic T cells expressing the antigens, leading to elimination of HIV-infected cells. However, the therapeutic effects of direct administration of bNAbs or bNAb-derived bispecific molecules are often compromised by their relatively short serum half-lives *in vivo* (45, 46).

Recent advances in synthetic biology have enabled innovative engineering strategies for cell therapy (47). By utilizing a synthetic Notch (synNotch) receptor system, which allows for inducing customized immune cell responses (48, 49), we developed novel bifunctional T cells that can controllably produce anti-HIV-1 bNAb and BiTE upon recognition of the viral antigen Env. This system integrates both local and systemic effects of bNAb and BiTE, offering a distinctive advantage in HIV treatment. The local induction of bNAb can promptly counteract viruses released by latency-reactivated reservoir cells, thereby preventing potential secondary infections (50). The BiTE molecule mediates the cytotoxic activity of engineered and neighboring CD8^+^ T cells against target cells in a proximal-to-distal manner (49, 51). Meanwhile, the systemic circulation of these effector molecules can reduce cell-free viruses and is anticipated to penetrate difficult-to-reach anatomical sites.

Here, we demonstrate that cells with the engineered synNotch receptor can respond to the Env proteins displayed on the surface of target cells or viral particles. Human primary CD8^+^ T cells equipped with the synNotch circuit can effectively reduce latency-reactivated cells and suppress viral replication without the risk of increasing susceptibility to viral infection in the engineered cells. These results suggest that the synNotch-T cells developed are promising candidates for AIDS therapy with the potential for future clinical applications.

## RESULTS

### Construction of a synNotch receptor for specific recognition of HIV-1 Env

**Fig 1:**
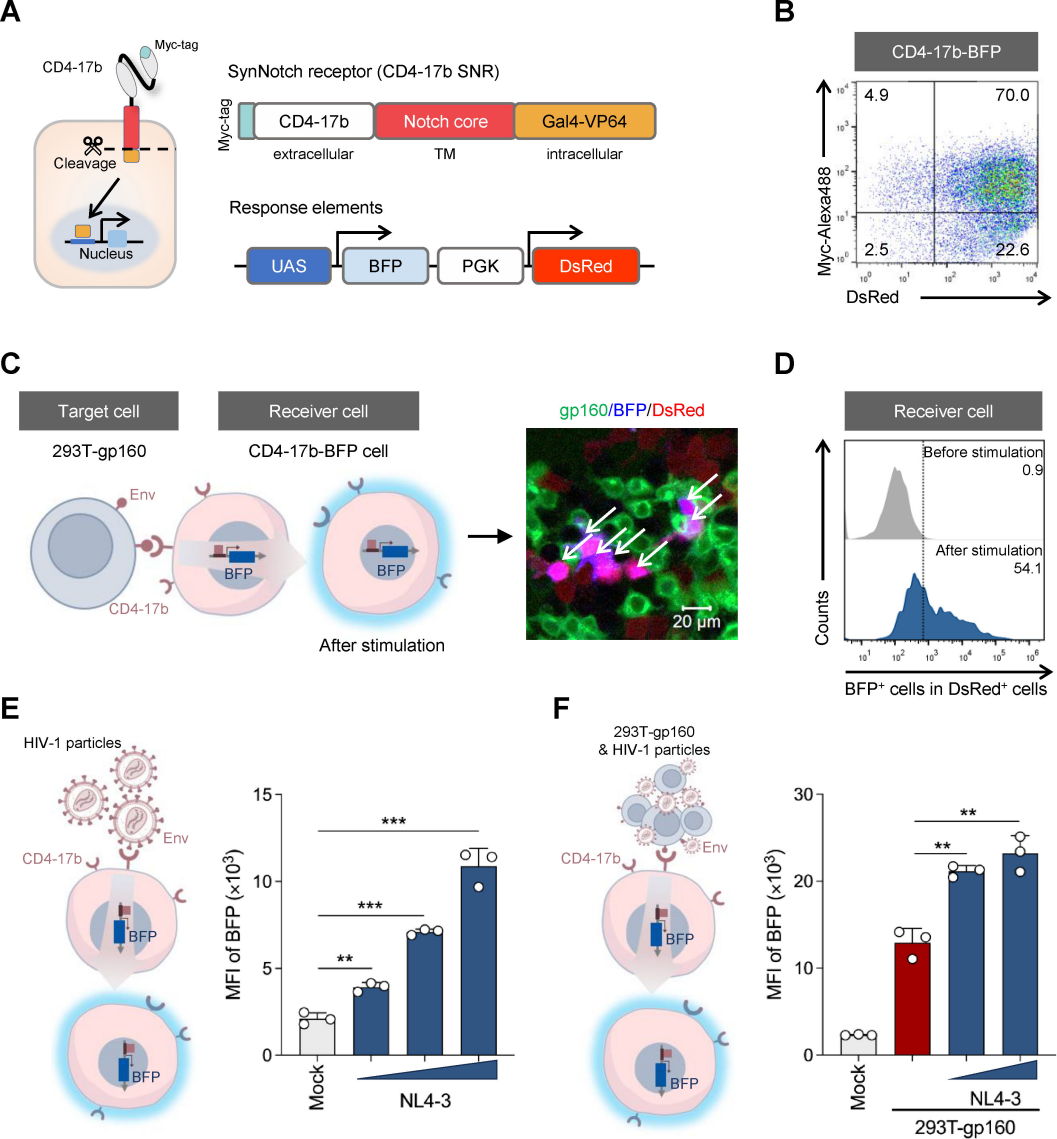
Engineered cells with a CD4-17b SNR can sense the Env-presenting cells and virions. (**A**) Schematic design of a CD4-17b-BFP cell. The CD4-17b molecule (Myc-tagged) targeting HIV-1 Env was used as the extracellular structural domain to bind to the Notch core and the cytoplasmic transcription factor Gal4-VP64 to generate the required synNotch receptor. Upon stimulation by the Env antigen, the detached transcription factor enters the nucleus and binds to the upstream activating sequence (UAS) of the regulated element, triggering the expression of BFP. The response element vector also carries a constitutive DsRed reporter for cell population analysis and purification. (**B**) The dual-positive rate of CD4-17b-BFP cells. Positive CD4-17b-BFP cells were measured by detecting both DsRed fluorescence and CD4-17b SNR stained with an anti-Myc antibody through flow cytometry, where the anti-Myc antibody was labeled by a secondary antibody conjugated to Alexa Fluor 488. (**C**) Expression of BFP in CD4-17b-BFP cells in response to 293T-gp160 cells. Left, schematic representation of CD4-17b-BFP cells reacting with the target cells. The immunofluorescence image (right) was taken after 48 h of co-culture of CD4-17b-BFP cells (red) with 293T-gp160 cells (green). An anti-Env 3B3 monoclonal antibody and the secondary antibody conjugated to Alexa Fluor 488 were used to detect 293T-gp160 cells. BFP and DsRed dual-positive cells are indicated by white arrows. The scale bar is 20 µm. (**D**) Flow cytometry analysis of the percentage of BFP-positive cells in the DsRed-positive CD4-17b-BFP cells described in panel C. (**E, F**) Flow cytometry analysis of the expression of BFP in CD4-17b-BFP cells (gated by DsRed^+^ expression) after stimulation with gradient virus particles without (**E**) or with (**F**) the addition of 293T-gp160 cells for 24 h, reported as mean fluorescence intensity (MFI). HIV-1_NL4-3_ particles were added at 0.05 µg (only in panel E), 0.25 µg, or 1.25 µg of p24. Mock, no virus or target cells added. In panels E and F, three independent experiments were performed and the error bars depict the standard deviation (SD). **, *P* < 0.01; ***, *P* < 0.001 (unpaired Student’s *t*-test).

The common structure of a synNotch receptor contains an extracellular antigen recognition domain, the notch core, and an intracellular structural domain of transcription factors (48). To achieve the conservative recognition of the Env antigen of different HIV-1 strains, an optimized bispecific binding molecule CD4-17b, which contains the first two extracellular structural domains (D1D2) of human CD4 and the scFv of an HIV-1 binding antibody 17b (52, 53), was used as the extracellular recognition domain of the synNotch receptor (SNR) (Fig. 1A). To determine the response capacity of the CD4-17b SNR to HIV-1 Env, we constructed a response element structure regulated by the synNotch receptor, which contains the blue fluorescent protein (BFP) as the induced reporter product and a constitutively expressed DsRed fluorescent protein driven by a PGK promoter for identifying the cells with the inserted response elements (Fig. 1A). This response pathway could be activated by the release of the transcription activator Gal4-VP64 upon antigen binding (48, 49) and was tested in human embryonic kidney (HEK) 293T cells (referred to as CD4-17b-BFP cells) by co-transducing the cells with lentiviruses carrying the CD4-17b SNR and the response elements, respectively. Flow cytometry analysis demonstrated the transduction efficiencies of CD4-17b SNR and the response elements in 293T cells, and the dual-positive rate was about 70% (Fig. 1B). Co-incubation of the CD4-17b-BFP cells with 293T-derived target cells expressing HIV-1_NL4-3_ Env glycoproteins (293T-gp160 cells, Fig. S1A and B) triggered the expression of BFP in CD4-17b-BFP cells (Fig. 1C). Approximately 54.1% of the DsRed-positive cells could express BFP after stimulation with the target cells (Fig. 1D), indicating that the CD4-17b synNotch pathway successfully responded to the HIV-1 Env proteins expressed on the cell surface.

Recent studies have shown that the viral antigens on hepatitis B virus (HBV) particles and the ligands displayed on the surface of microbeads can also activate the corresponding synNotch pathways (54–56). This suggests that HIV-1 particles, which are similarly enveloped viruses with larger particle sizes than HBV, may have the ability to directly activate the CD4-17b synNotch pathway. To test this assumption, we co-incubated CD4-17b-BFP cells with different doses of HIV-1_NL4-3_ virus particles. The expression level of BFP was increased with the amounts of virus particles in a dose-dependent manner (Fig. 1E). Furthermore, the co-addition of the target cells and viral particles to the receiver cells resulted in higher expression levels of BFP than the addition of the target cells only (Fig. 1F). Together, these results suggest that the CD4-17b SNR can be activated by the HIV-1 Env antigen presented on both viral particles and the surface of target cells.

### T cells equipped with the synNotch circuits can produce functional anti-HIV-1 bNAb and BiTE in response to sensitization

To develop genetically engineered T cells with dual functions of mediating neutralization of HIV-1 virions and cytotoxicity of infected cells, we replaced the BFP reporter gene with the genes of bNAb and/or BiTE. Hundreds of highly efficient monoclonal bNAbs have been identified and characterized in neutralization assays (46, 57). We first chose three highly broad and potent bNAbs, VRC01 (58), N6 (59) (both targeting the CD4-binding site), and 10E8v4 (gp41 membrane-proximal external region, MPER) (60, 61), that bind conserved epitopes on the HIV-1 Env glycoprotein, and one bispecific bNAb (BiAb), PGDM1400-10E8v4 (targeting both the variable regions 1 and 2 glycan loop and MPER) (62), to test their response efficiency in 293T cells (Fig. S2A). After stimulation with 293T-gp160 cells, the cells engineered with CD4-17b SNR and the VRC01 response element demonstrated the highest dual-positive transduction efficiency and antibody secretion level, while those in the BiAb group were the lowest (Fig. S2B and C), possibly due to the limitation in transduction efficiency of longer genes from lentiviral vectors. Therefore, VRC01 was selected as the response element for the following study.

**Fig 2:**
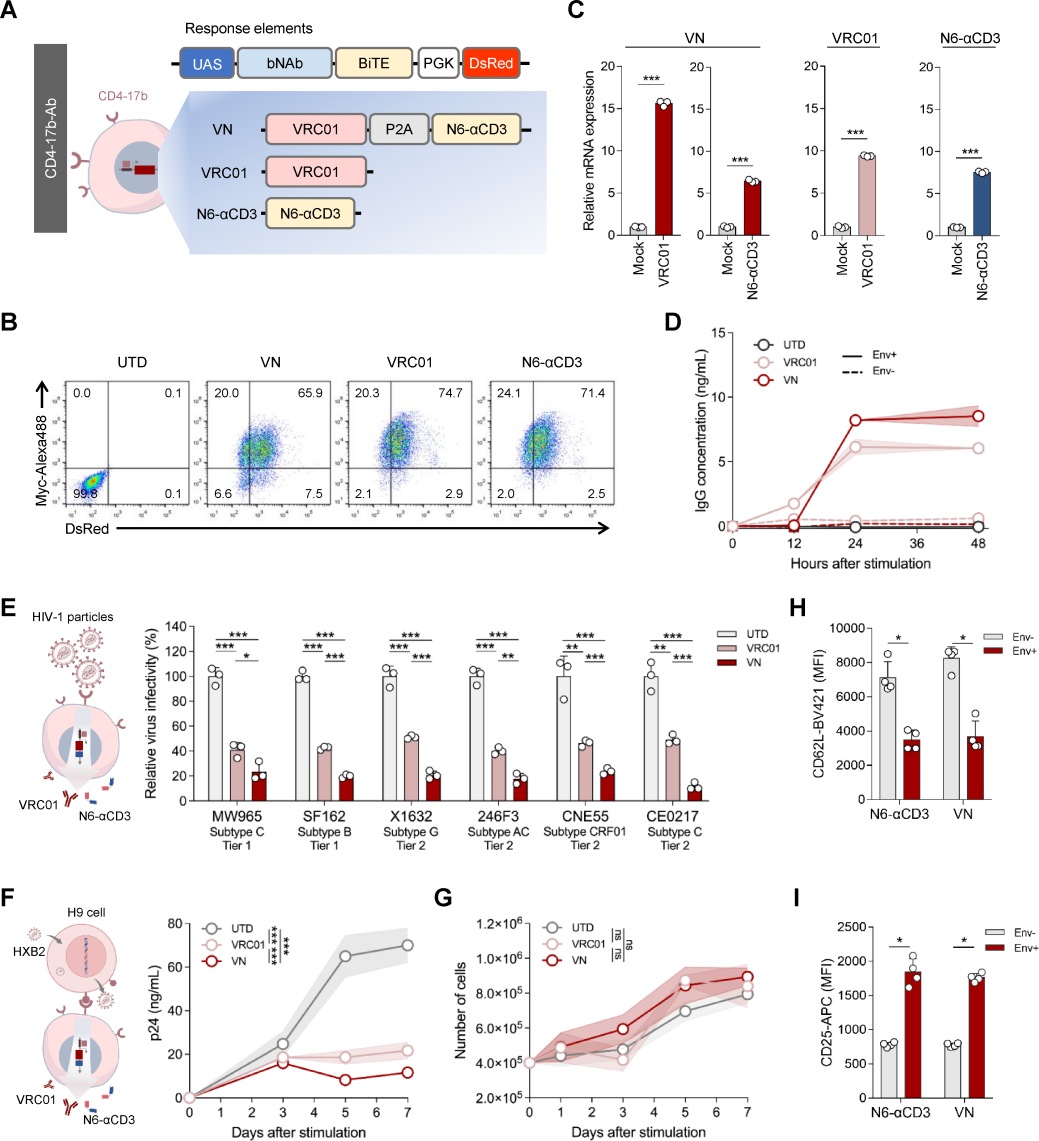
Functional anti-HIV-1 bNAb and BiTE can be produced after activation of the Jurkat T cells equipped with the synNotch circuits. (**A**) Schematic design of the response elements of the CD4-17b-Ab synNotch circuits. (**B**) The dual-positive rate of CD4-17b-Ab Jurkat cells, as determined by flow cytometry at 7 days after FACS purification, was analyzed through the detection of both Myc-tagged CD4-17b SNR and DsRed. Jurkat cells were engineered with CD4-17b SNR and the response element encoding for VN, VRC01, or N6-αCD3. UTD, untransduced. (**C**) The Ab expression of the CD4-17b-Ab cells in panel B after being stimulated with 293T-gp160 for 24 h was determined by RT-qPCR probed to VRC01, N6-αCD3, or both genes. Mock, stimulated with 293T cells. (**D**) Time course kinetics of VRC01 IgG secretion from CD4-17b-Ab or UTD Jurkat cells stimulated with 293T-gp160 (Env+, solid lines) or 293T (Env−, dashed lines) cells. (**E**) After 24 h of co-incubation of CD4-17b-Ab cells or UTD cells with pseudoviruses with the Env proteins from multiple HIV-1 strains, changes in the supernatant virus infectivity were detected by TZM-bl cells. (**F**) The p24 viral replication curves were measured with the co-culture supernatants of H9-HXB2 cells and CD4-17b-Ab cells or UTD cells. H9-HXB2 cells are H9 cells infected with HIV-1_HXB2_ for 3 days. (**G**) The cell number of each group in panel F was monitored within the co-culture course. (**H, I**) Flow cytometry analysis of CD62L (**H**) and CD25 (**I**) T cell activation markers on CD4-17b-Ab Jurkat cells (gated by DsRed^+^ expression) co-incubated with 293T-gp160 cells for 24 h, reported as mean fluorescence intensity (MFI). In panels C to I, three (C to G) or four (**H and I**) independent experiments were performed and the error bars depict SD. In panels C and E to I, statistical analysis was performed by unpaired Student’s *t*-test (**C and E**), two-way repeated measures analysis of variance (**F and G**), or unpaired Mann-Whitney U-test (**H and I**). *, *P* < 0.05; **, *P* < 0.01; ***, *P* < 0.001; ns, not significant.

Then, we generated a BiTE by fusing the N6 scFv with an anti-human CD3ε scFv (N6-αCD3). The bifunctional synNotch circuit expresses both VRC01 and N6-αCD3 (VN) controlled by the CD4-17b SNR and was evaluated in Jurkat T cells (Fig. 2A). CD4-17b-VRC01 and CD4-17b-N6-αCD3 circuits were also constructed as the single-functional controls for the CD4-17b-VN circuit to determine whether VRC01 and N6-αCD3 function as expected. The transduced cells were purified by fluorescence-activated cell sorting (FACS), and the dual-positive rate of the cells was assessed after purification (Fig. 2B). The mRNA levels of the induced products, which represent the activation of the circuits, were detected by reverse transcription-quantitative PCR (RT-qPCR) after the engineered Jurkat cells sensed 293T-gp160 cells (Fig. 2C). Furthermore, the levels of Env-primed antibodies (Abs) secreted by the engineered cells were quantified by detecting the Fc of VRC01 using an enzyme-linked immunosorbent assay (ELISA). The CD4-17b-Ab cells showed very low basal activation when the sender cells did not display the Env proteins, while efficiently secreting the Abs after incubation with 293T-gp160 cells (Fig. 2D).

To confirm whether HIV-1 particles could activate the engineered T cells and whether the Abs produced by the cells could in turn neutralize the viral particles, we measured the infection rate of the supernatant viruses using TZM-bl cells following co-incubation of HIV-1 particles of different subtypes and tier with the engineered cells. The infection rate of the four tier 2 and two tier 1 viruses significantly decreased after exposure to CD4-17b-VRC01 and CD4-17b-VN cells compared to the untransduced (UTD) cell group (Fig. 2E), suggesting the effective activation of the engineered T cells by the viral particles and the subsequent neutralization by the Abs. A better performance of CD4-17b-VN cells than CD4-17b-VRC01 cells was observed (Fig. 2E), which may result from the higher Ab-secreting level of CD4-17b-VN cells (Fig. 2D) and the cooperated neutralization effects by both VRC01 and N6-αCD3 (63).

Next, we evaluated the long-term inhibitory effect of CD4-17b-Ab cells on HIV-1 replication by preventing viral infection by the Abs, using HIV-1_HXB2_-infected H9 (H9-HXB2) cells as the target cells (Fig. S2D). Three days post-infection, H9-HXB2 cells were incubated with the CD4-17b-Ab cells which can be activated by both the infected cells and newly produced virions. The viral replication level in the co-culture system was quantified by HIV-1 p24 ELISA. HIV-1_HXB2_ replication in H9 cells was effectively suppressed by both CD4-17b-VRC01 and CD4-17b-VN cells over 7 days of incubation (Fig. 2F) without difference in the cell growth (Fig. 2G), and CD4-17b-VN cells showed higher suppression efficacy.

To determine the efficiency of T cell activation by the Env-primed N6-αCD3, we evaluated the activation properties of CD4-17b-VN and CD4-17b-N6-αCD3 cells as the engineered Jurkat cells are CD3-expressing cells. T cell activation leads to downregulation of cell surface CD62L and upregulation of CD25 (64, 65). The downregulated CD62L and upregulated CD25 on the surface of the two engineered cells indicated that N6-αCD3 secreted in the presence of target cells could mediate cell activation (Fig. 2H and I). These results suggest that the CD4-17b-Ab circuits in Jurkat T cells can produce functional bNAb and BiTE in an Env-controlled manner.

### The Env-primed BiTE has paracrine-mediated redirected killing activity against HIV-1 infected and latency-reactivated cells

**Fig 3:**
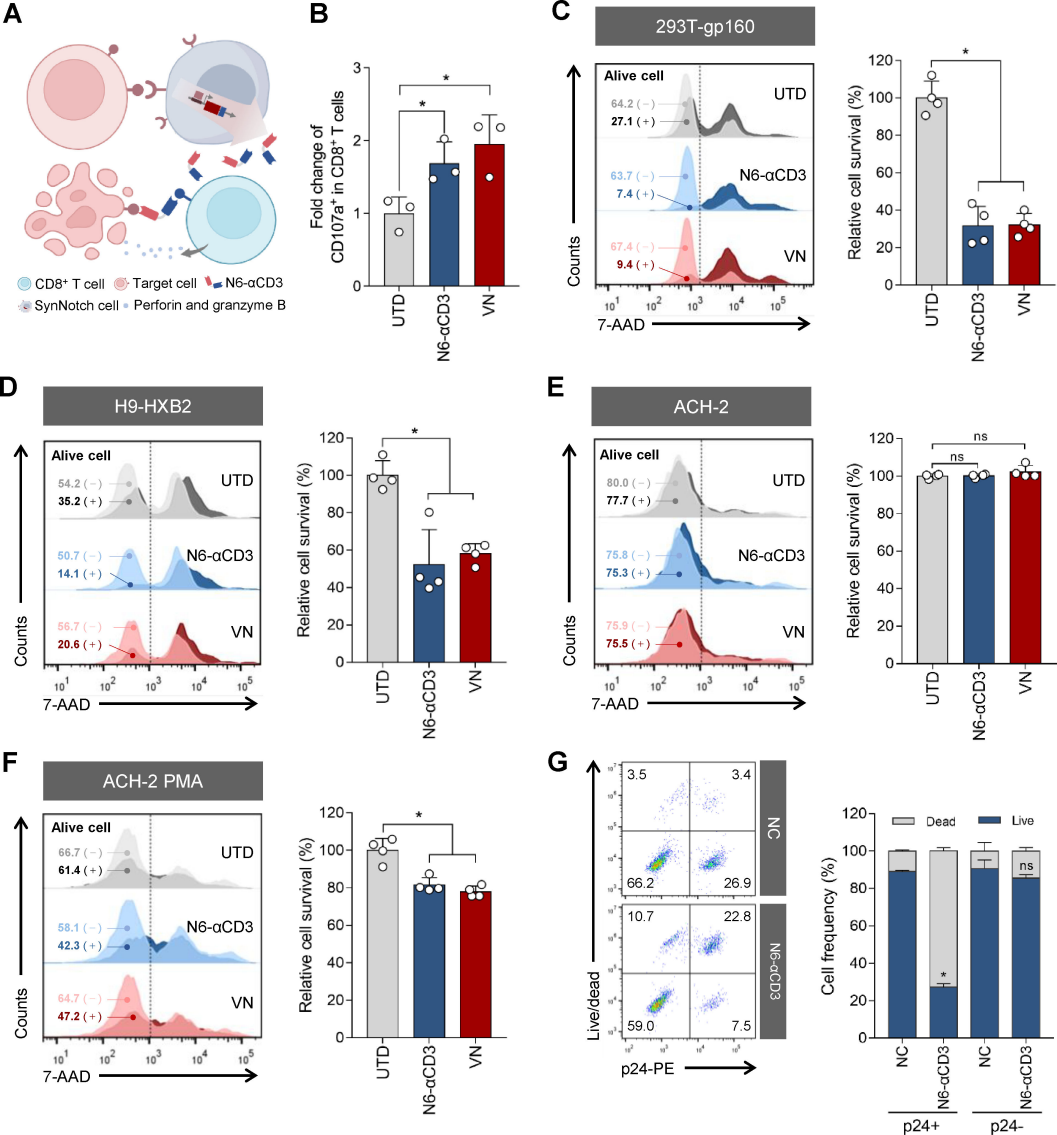
Env-driven BiTE production from the engineered T cells mediates bypass activation and cytotoxicity of CD8^+^ T cells. (**A**) Schematic diagram of Env-driven N6-αCD3 mediating the killing of target cells by CD8^+^ T cells in a paracrine manner. (**B**) Flow cytometry analysis of CD107a expression in the CD8^+^ T cell population mediated by Env-driven N6-αCD3 at 24 h post-co-culture. The CD107a level in the UTD group was set as the reference control. (C to F) 293T-gp160 cells (**C**), HXB2-infected H9 cells (**D**), ACH-2 cells (**E**), or PMA-activated ACH-2 cells (**F**) were co-cultured with CD4-17b-Ab or UTD cells at a 1:1 ratio for 20 h. Then, the percentage of viable target cells was analyzed by flow cytometry before and after 6 h of incubation with CD8^+^ T cells. The representative histograms from four independent cytotoxicity assays were selected for demonstration (left). Light and dark histograms indicate target cell viability before (−) and after (+) the addition of CD8^+^ T cells. The relative percentage of survival cells (right) was calculated by dividing the percentage of viable cells remaining after the addition of CD8^+^ T cells by the percentage before addition, and then expressing this relative to the survival rate in the UTD group, which was set as 100%. (**G**) HXB2-infected CD4^+^ T cells were stained with a far-red live/dead cell staining reagent followed by PE staining of intracellular p24. The cells were then co-cultured with CD8^+^ T cells at a 1:1 ratio for 6 h with or without the addition of 10 ng purified N6-αCD3 protein. NC, negative control. The proportion of surviving cells within p24-positive and -negative populations was analyzed by flow cytometry (left). The relative percentages of living and dead cells were calculated separately for p24-positive and -negative populations (right). In panels B to G, three (**B**) or four (C to G) independent experiments were performed and the error bars depict SD. Statistical analysis was performed by unpaired Student’s *t*-test (**B**) or Mann-Whitney U test (C to G). *, *P* < 0.05; ns, not significant.

Compared with anti-HIV CAR-T cells, which typically execute their killing function autonomously after activation, synNotch-T cells can mediate a broader cytotoxic T cell response by secreting BiTEs to activate both themselves and surrounding naïve T cells. From our results (Fig. 2G), CD4-17b-VN Jurkat cells secreting N6-αCD3 did not have the killing capacity on target cells. To investigate whether N6-αCD3 secreted by CD4-17b-Ab cells can bridge cytotoxic T cells to Env-expressing cells to induce killing of the infected cells, we added human primary CD8^+^ T lymphocytes into the co-culture system of the target cells and CD4-17b-Ab Jurkat cells (Fig. 3A). Flow cytometry analysis of CD107a on the surface of co-incubated CD8^+^ T cells demonstrated that the Env-primed N6-αCD3 induced degranulation of the CD8^+^ T cells in the presence of 293T-gp160 cells (Fig. 3B). By measuring the proportion of the target cell populations before and after the addition of CD8^+^ T cells, both CD4-17b-N6-αCD3 and CD4-17b-VN cells significantly mediated the lysis of about 70% of 293T-gp160 cells and 50% of HIV-1_HXB2_-infected H9 cells (Fig. 3C and D).

To further investigate the performance of CD4-17b-Ab cells in response to HIV-1 latently infected cells, ACH-2 cells integrated with one proviral copy of latent HIV-1_LAV_ (66) were used as the target cells in the presence or absence of the phorbol 12-myristate 13-acetate (PMA) treatment. The co-culture of ACH-2 cells with CD4-17b-Ab cells and CD8^+^ T cells did not result in any notable killing (Fig. 3E). However, when the cells were pre-activated with PMA for 24 h to enable the expression of Env (Fig. S3A), about 20% of ACH-2 cells in the N6-αCD3 and VN groups were killed by the addition of CD8^+^ T cells (Fig. 3F).

To verify that the cytotoxic effect mediated by N6-αCD3 is specifically targeted to HIV-infected (p24-positive) cells, we infected human primary CD4^+^ T lymphocytes with HIV-1_HXB2_ and subsequently assessed the survival of both p24-positive and -negative cells in the presence of purified N6-αCD3 protein and CD8^+^ T cells at 2 days post-infection. The results demonstrated that CD8^+^ T cells specifically killed p24-positive cells in the presence of N6-αCD3, as evidenced by a significant reduction in the proportion of viable p24-positive cells compared to those in control groups without N6-αCD3 (Fig. 3G). Taken together, these results indicate that the CD4-17b-Ab cells can effectively reduce the target cell population by the secreted N6-αCD3 in a paracrine-mediated manner.

### Human CD8^+^ T lymphocytes engineered with the bifunctional synNotch circuit can suppress viral replication and kill latency-reactivated CD4^+^ T cells directly

**Fig 4:**
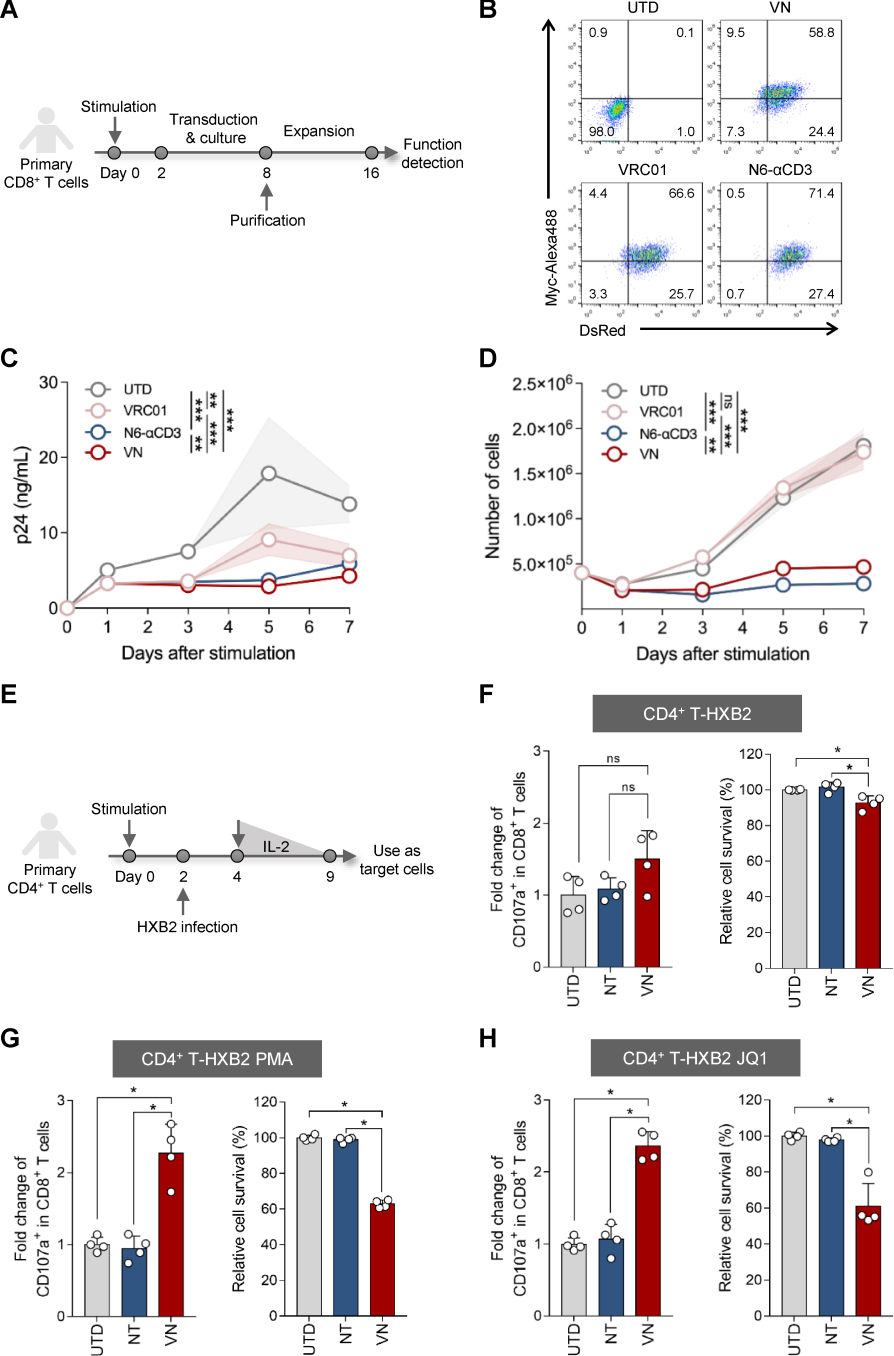
CD8^+^ T cells equipped with the bifunctional synNotch circuit can control viral replication and kill HIV-1 latency-reactivated cells. (**A**) Schematic diagram of the generation of CD8^+^ T cells equipped with the synNotch circuits. (**B**) The dual-positive rate of CD4-17b-Ab CD8^+^ T cells was determined by flow cytometry 7 days after FACS purification. (**C**) The p24 viral replication curves were measured with the co-culture supernatants of H9-HXB2 cells and CD4-17b-Ab CD8^+^ T cells or UTD cells. (**D**) The cell number of each group in panel C was monitored within the co-culture course. (**E**) Establishment of an HIV latent infection model using human primary CD4^+^ T cells. CD4^+^ T cells were first activated and expanded with anti-CD3 and anti-CD28 antibodies for 2 days. The cells were then infected with HIV-1_HXB2_ and maintained with gradually decreasing concentrations of interleukin-2 (IL-2) for 5 days from day 2 post-infection to establish latency. (F to H) The survival of HIV-1_HXB2_ latently-infected CD4^+^ T cells, without (**F**) or with 24 h of PMA (**G**) or JQ1 (**H**) pre-treatment, was analyzed by flow cytometry before and after 24 h of incubation with engineered or UTD CD8^+^ T cells. Anti-CD19 SNR-transduced cells severed as a non-targeting (NT) control. CD107a expression on the engineered or UTD CD8^+^ T cells was analyzed by flow cytometry following stimulation with the target cells (left). The relative percentage of survival cells (right) was calculated as described in the legend of Fig. 3. In panels C, D, and F to H, three (**C and D**) or four (F to H) independent experiments were performed and the error bars depict SD. Statistical analysis was performed by repeated measures analysis of variance (**C and D**) or Mann-Whitney U-test (F to H). *, *P* < 0.05; **, *P* < 0.01; ***, *P* < 0.001; ns, not significant.

To demonstrate that cytotoxic T cells customized with the bifunctional synNotch circuit are a potential tool for AIDS therapy, we engineered primary CD8^+^ T lymphocytes with the CD4-17b-Ab circuits (Fig. 4A and B). The bifunctional CD8^+^ T cells should be able to simultaneously reduce HIV-1-infected cells and cell-free virus infection. Therefore, we investigated the suppression effect of CD4-17b-VN cells on long-term viral replication and the killing efficiency of latency-reactivated cells, respectively. First, we compared the effect of CD4-17b-VN cells on suppressing long-term viral replication with that of the single-functional CD4-17b-VRC01 and CD4-17b-N6-αCD3 cells by co-culturing them with H9-HXB2 cells, respectively. The results demonstrated a significant reduction in viral p24 levels in the culture supernatants of the VRC01, N6-αCD3, and VN groups compared with that of the UTD group over the 7 days of co-culture (Fig. 4C). CD4-17b-VN cells showed a better suppression effect than both CD4-17b-VRC01 and CD4-17b-N6-αCD3 cells and superior ability in killing the infected H9 cells as CD4-17b-N6-αCD3 cells (Fig. 4D).

To enhance the precision of evaluating the cytotoxic capabilities of CD4-17b-Ab CD8^+^ T cells, we established a latent infection model using human primary CD4^+^ T lymphocytes (Fig. 4E) (67, 68). The Env expression, p24-positive cell proportion, and cell viability in the infected CD4^+^ T cells was confirmed after 2 days of infection, during latency (at day 9), and upon re-activation (after PMA or JQ1 treatment) via Western blotting and flow cytometry (Fig. S3B and C). Additionally, a similar synNotch receptor, using anti-CD19 scFv as the extracellular recognition domain (48), was included as a non-targeting (NT) control for co-transduction of CD8^+^ T cells expressing the VN response element. Then, CD4-17b-VN, NT, or UTD CD8^+^ T cells were co-cultured with HIV-1_HXB2_ latently infected CD4^+^ T (CD4^+^ T-HXB2) cells with or without PMA or JQ1 pre-treatment. After 24 h of co-culture, approximately 40% of treated CD4^+^ T-HXB2 cells were eliminated by the bifunctional CD8^+^ T cells, accompanied by an upregulation of CD107a expression. In contrast, no CD107a upregulation or specific killing was observed when the bifunctional CD8^+^ T cells were co-cultured with unactivated CD4^+^ T-HXB2 cells (Fig. 4F through H; Fig. S3D to F). Notably, the proportion of p24-positive cells among those treated by PMA and JQ1 was approximately 45% (Fig. S3C), suggesting that CD4-17b-VN cells specifically killed more than 85% of the reactivated cells. These results indicate that the bifunctional synNotch-T cells can efficiently control viral replication and reduce reactivated HIV-1 latency cells.

### The CD4-17b SNR does not increase the unwanted infection risk of the engineered cells by HIV-1

**Fig 5:**
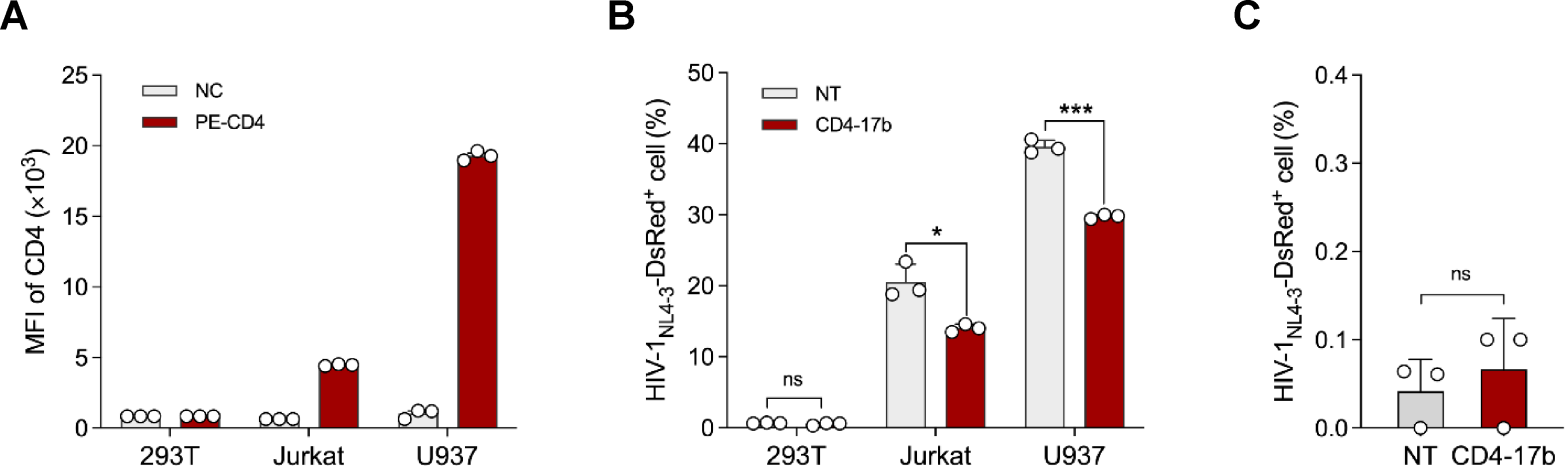
HIV-1 infection susceptibility of the cells engineered with CD4-17b SNR. (**A**) Flow cytometry detection of CD4 expression on the surface of 293T, Jurkat, and U937 cells. NC, PE-CD4 antibody-unstained cells as the negative control. (**B, C**) Flow cytometry detection of the infection efficiency of HIV-1_NL4-3_-DsRed in 293T, Jurkat, and U937 cells (**B**) and CD8^+^ T cells (**C**) equipped with CD4-17b SNR or anti-CD19 NT SNR after being challenged with the virus for 3 days. Three independent experiments were performed and the error bars depict SD. *, *P* < 0.05; ***, *P* < 0.001; ns, not significant (unpaired Student’s *t*-test).

A major concern about CD4 molecule-based elements used for the HIV-1 antigen recognition receptors is that they may render the engineered cells susceptible to viral infection. It has been verified that CD4-17b CAR does not have an undesired function as HIV-1 entry receptors (53). We further tested this possibility when CD4-17b was used as the recognition domain of the synNotch receptor. Three cell lines were selected to test the possibility: 293T cells lacking CD4 expression, Jurkat T cells with low CD4 expression, and a myeloid-derived monocyte cell line U937 with high CD4 expression (Fig. 5A). These cells were transduced with CD4-17b SNR and then challenged with HIV-1_NL4-3_ carrying a DsRed reporter gene (HIV-1_NL4-3_-DsRed). Flow cytometry analysis showed that the infection rate of HIV-1_NL4-3_-DsRed in the CD4-17b SNR group was lower than that in the NT SNR group in Jurkat and U937 cells (Fig. 5B), suggesting that CD4-17b SNR may compete with the CD4 receptors on these cells in binding with the virus.

Subsequently, the same experiments were performed in CD8^+^ T cells. As low as observed in 293T cells (Fig. 5B), the viral infection rate between the CD4-17b and NT SNR groups showed no significant difference (Fig. 5C). These results indicate that CD4-17b SNR does not mediate undesired HIV-1 entry activity.

## DISCUSSION

Despite decades of effort, AIDS remains one of the most prevalent and detrimental infectious diseases worldwide. Only a limited number of AIDS patients have been declared cured by undergoing allogeneic hematopoietic stem cell transplantation utilizing the cells from CCR5Δ32/Δ32 donors (69–72). While implementing this strategy on a large scale may not be currently feasible for the treatments, these studies offer hope that enhanced immune system functionality could potentially lead to the eradication or functional cure of AIDS.

In the current study, we employed a synthetic biology approach to engineer T cells, which enables the coordination of humoral and cellular immune responses customized for anti-HIV-1. Before our research, the application of the synNotch receptor system in anti-HIV immune cell therapy had not been explored. The superior performance of the synNotch receptor platform has been demonstrated in the engineering of anti-cancer T cells, not only for antibody secretion (49). For AIDS therapy, the synNotch-T cells designed by us have several advantages over conventional HIV-specific CAR-T cells: first, the CD4-17b SNR can respond to both HIV-1 virions and infected cells, like what was observed in response to HBV antigens (54), which increases the opportunity of the engineered T cells to be activated *in vivo*. Second, the expression of bNAbs and BiTEs by cells can prolong their serum half-lives. Furthermore, in contrast to the previously employed continuous expression strategies utilized in Ab-secreting CAR-T cells by fusing bNAbs to the end of the costimulatory domain of CAR (73, 74), the regulation of antibody secretion by the synNotch-T cells is driven by HIV-1 Env stimulation, thereby ensuring precise control over the immune responses. Third, the engineered T cells demonstrated both autocrine- and paracrine-mediated cytotoxic effects on target cells through the secretion of BiTE molecules, thereby inducing both local and systemic responses to antigenic stimulation at HIV-1 infection sites and reservoirs. Previous studies on anti-cancer CAR-T cells have shown that BiTE molecules are highly potent and can achieve significant therapeutic effects even at relatively low concentrations (75, 76). Additionally, the bNAb molecules produced by the engineered cells, upon binding to latency-reactivated reservoir cells, are expected to immediately prevent potential secondary infections in the local environment. These molecules can also disseminate systemically throughout the body to reduce cell-free viral loads.

A feature of bNAb-derived BiTEs is that they also have neutralization activity against various HIV-1 strains (63). Although the CD8^+^ T cells engineered with the CD4-17b-N6-αCD3 and CD4-17b-VN circuits demonstrated comparable efficacy in suppressing viral replication and cytotoxicity (Fig. 4C and D) in our *in vitro* model, the neutralizing potency of bNAb-derived BiTEs or DARTs is generally lower than the original bNAbs (63, 77). Therefore, incorporating a bNAb into the design of bifunctional synNotch-T cells is necessary, particularly for the *in vivo* applications. In addition to the function of effectively neutralizing cell-free viruses by the Env-primed VRC01, it has been suggested that the output of VRC01 may also mediate antibody-dependent cellular cytotoxicity (ADCC) (78) and play a role in inhibiting cell-to-cell virus infection (79). Furthermore, modifying the Fc domain of VRC01 can enhance its activity in mediating ADCC (80, 81), enabling the generation of tri-functional synNotch-T cells. More importantly, this platform is modular as VRC01 can be replaced by other potent bNAbs, even BiAbs or trispecific antibodies, although the practical application remains challenging due to limitations associated with available long-gene delivery technology. In our results, both gene transduction and secretion were compromised for the BiAb PGDM1400-10E8v4 (Fig. S2B and C). Therefore, improving T cell gene editing efficiency represents a critical need for future cell therapy endeavors (82), especially for the synNotch receptor system which necessitates co-transduction of two types of lentiviruses.

The major limitation of this study is that although we have conceptually validated the design of the bifunctional synNotch-T cells, the safety and therapeutic effect of the cells were not evaluated within an *in vivo* environment. It has been demonstrated in many *in vivo* studies that T cells engineered with synNotch circuits are maintained in a more naïve-like memory T cell phenotyping which is more durable and less prone to exhaustion than the cells with CAR modification (83, 84). This finding supports the potential of our bifunctional synNotch-T cells to exhibit persistence and long-lasting effects *in vivo*, which is a crucial requirement for anti-HIV adoptive T cell therapy aimed at suppressing plasma viremia and eradicating viral reservoir (24). Furthermore, recent innovations, such as the development of more compact synNotch receptors exemplified by the SNIPR, offer superior modularity and programmability, enabling easier cell engineering and lower immunogenicity for clinical use (85). These advancements underscore the opportunity for future research to refine and adapt these methods, establishing our study as a critical foundational work for the clinical translation of these sophisticated cellular engineering approaches. Future directions for optimizing the synNotch-T cells emphasize enhancing receptor sensitivity to the viral antigens, employing more potent effector molecules, and incorporating multiple therapeutic functions while maintaining the output efficiency of these molecules.

Overall, our engineered T cells with CD4-17b SNR controlling the output of bNAb and BiTE can effectively mobilize host immune cells to combat the virus, aligning with the requirements of an ideal anti-HIV cell therapy strategy. Based on the evidence demonstrating that CD8^+^ T cells equipped with the bifunctional synNotch circuit can efficiently reduce latency-reactivated cells and suppress viral replication *in vitro*, these cells are promising to be therapeutically used as part of a “shock and kill” regimen (86) when combined with LRAs toward achieving a functional cure for AIDS.

## MATERIALS AND METHODS

### Isolation and culture of human primary T lymphocytes

Peripheral blood mononuclear cells (PBMCs) were isolated from the healthy donor blood by Lymphoprep density gradient separation and SepMate tubes (Stemcell Technologies, Vancouver, Canada). Primary CD8^+^ and CD4^+^ T lymphocytes were obtained from PBMCs by magnetic selection using a human CD8^+^ T cell isolation kit (Miltenyi Biotec, Bergisch Gladbach, Germany) and CD4 microbeads (Miltenyi Biotec), respectively. CD8^+^ and CD4^+^ T cells were cultured at a density of 10^6^ cells/mL in a human T cell medium consisting of X-VIVO 15 (Lonza, Basel, Switzerland), 10% fetal bovine serum (FBS), 50 UI/mL recombinant human interleukin-2 (IL-2) (PeproTech, Cranbury, NJ), and 10 ng/ml IL-7 (PeproTech) supplemented with 100 IU/mL penicillin and 100 IU/mL streptomycin at 37°C in 5% CO_2_. Before lentiviral transduction or viral infection, CD8^+^ and CD4^+^ T cells were stimulated for 2 days with an anti-CD3 antibody at 2.5 µg/mL and an anti-CD28 antibody at 2.5 µg/mL (BioLegend, San Diego, CA).

### Cell line culture

HEK-293T cells (CRL-11268) were purchased from the American Type Culture Collection (Manassas, VA). TZM-bl cells, human H9 and Jurkat T cells, myeloid-derived U937 monocytes, and HIV-1 latently infected ACH-2 cells were obtained from the National Institutes of Health, HIV Reagent Program (NIH-HRP). Expi293F cells were purchased from Thermo Fisher Scientific (Waltham, MA). 293T and TZM-bl cells were cultured in Dulbecco’s modified Eagle’s medium (DMEM) containing 10% FBS at 37°C in 5% CO_2_. H9, Jurkat, U937, and ACH-2 cells were maintained in Roswell Park Memorial Institute (RPMI) 1640 medium with 10% FBS at 37°C in 5% CO_2_. Expi293F cells were cultured in Expi293 Expression Medium (Thermo Fisher Scientific) at 37°C in a humidified incubator with 8% CO_2_, at a shaking speed of 120 rpm. All cell culture media contained 100 IU/mL penicillin and 100 IU/mL streptomycin.

### Preparation of target cells

The 293T-gp160 cell line was generated by lentiviral transduction of 293T cells with HIV-1 gp160 and purified by FACS to ensure the positive rate above 90% using the BD INFLUX cell sorter (BD Biosciences, San Jose, CA). H9-HXB2 cells were generated by infecting H9 cells with HIV-1_HXB2_ at 10^−1^ 50% tissue culture infectious dose (TCID_50_)/cell, 3 days prior to co-culture with CD4-17b-Ab cells or UTD cells. To establish a latent infection in human primary CD4^+^ T cells, purified CD4^+^ T cells were activated with the anti-CD3 and anti-CD28 antibodies for 2 days. The cells were then infected with 10^−1^ TCID_50_/cell HIV-1_HXB2_ and maintained with gradually decreasing concentrations of IL-2 over 5 days from day 2 post-infection to establish latency. Latently infected CD4^+^ T cells and ACH-2 cells were stimulated with 5 ng/mL PMA (Beyotime, Shanghai, China) or 10 µM JQ1 (TargetMol, Boston, MA) for 24 h, followed by washing in phosphate buffered saline (PBS) before being co-cultured with CD4-17b-Ab cells or UTD cells.

### Construct design

The synNotch receptor vector expressing anti-CD19 scFv as the extracellular recognition domain to fuse to the notch core domain and the intracellular Gal4-VP64 transcription factor (48) and the response element vector expressing the BFP reporter were purchased from Addgene (Watertown, MA). The CD4-17b SNR vector was obtained by replacing the anti-CD19 scFv gene with a Myc-tagged gene fragment of CD4-17b (53). The response element vectors expressing the heavy and light chains of VRC01, N6, 10E8v4, BiAb, N6-αCD3 scFv, or VN were generated by replacing the BFP reporter gene with the corresponding encoding genes. To facilitate the identification of transduced cells, all response element vectors contained a PGK promoter constitutively driving the expression of DsRed.

### Lentivirus production and cell transduction

The HIV-1 infectious molecular clones pNL4-3 and pHXB2 were provided by NIH-HRP. pNL4-3-DsRed was constructed by inserting the DsRed gene between the open reading frame of *env* and *nef*. Viruses were generated by transfection into 293T cells. To produce lentiviruses pseudotyped with pantropic vesicular stomatitis virus glycoprotein, 293T cells were transfected with the synNotch receptor vector or the response element vector and the viral packaging plasmids psPAX2 and pMD2.G (Addgene) at a mass radio of 4:3:1 using a jetPRIME transfection reagent (Polyplus-transfection, Illkirch, France). Viral supernatants were harvested at 48 h to 72 h post-transfection by centrifugation and passed through 0.22 µm filters. For transduction of 293T cells, the cells were co-infected with the lentiviruses generated from the CD4-17b SNR vector and the response element vector in six-well plates with 8 µg/mL of polybrene (Yeason, Shanghai, China) for 24 h. The transduced cells were subsequently passaged by ethylenediaminetetraacetic acid-free trypsin digestion to avoid ligand-independent Notch/synNotch activation (87). For the transduction of Jurkat cells and primary CD8^+^ T cells, the viral supernatants from transfected 293T cells were concentrated with a lentivirus concentration solution (GeneCopoeia, Rockville, MD) according to the manufacturer’s instructions, and the cells were then transduced as described previously (48). Polyclonal-engineered cell populations were stained for surface expression and then purified for both high receptor expression and high DsRed expression by FACS using the BD INFLUX cell sorter.

### SynNotch-driven BFP expression or antibody production

293T cells, Jurkat cells, and human CD8^+^ T cells were co-transduced with CD4-17b SNR and the response element vector expressing BFP or one of the Abs. Transduced cells (1 × 10^5^ or 2 × 10^5^ transduced cells were mixed with target cells in suspension at a 1:1 ratio or with HIV-1 particles and co-cultured in 96-well U-bottom plates for 24 h after centrifugation at 400 × *g* for 1 min. The co-cultured cells or the supernatants were harvested and detected for BFP expression using immunofluorescence microscopy or flow cytometry, or for antibody production using ELISA.

### RT-qPCR

RNA was extracted with a TRIzol reagent (TransGen Biotech, Beijing, China), and cDNA was synthesized using a TransScript All-in-One First-Strand cDNA Synthesis SuperMix kit containing random primers (TransGen Biotech). qPCR was performed with a TransStart Top Green qPCR SuperMix kit (TransGen Biotech) using the Bio-Rad CFX thermocycler and CFX Manager software. Target gene expression was normalized to the GAPDH using the 2^ΔΔCt^ method. Primer sequences for qPCR are provided in Table S1.

### ELISA

To detect antibody expression in cell supernatants, 96-well plates were coated with a goat anti-monkey IgG (H+L) secondary antibody (Novus Biologicals, Littleton, CO) in advance at 4°C overnight. On the next day, the plate wells were blocked with a PBS solution containing 5% bovine serum albumin for 2 h at 37°C, then rinsed three times with PBS containing 0.1% Tween-20 (PBS-T). Then, the supernatants containing antibodies were added to the wells and incubated at 37°C for 2 h. A known concentration of VRC01 protein (NIH-HRP) was serially diluted to generate a standard curve. Three sub-wells were set up for each sample. The plates were washed three times with PBS-T, followed by incubating with a horseradish peroxidase-conjugated anti-human IgG secondary antibody (Jackson ImmunoResearch, West Grove, PA) for 1 h at 37℃, then rinsed another three times with PBS-T, followed by reacting with a tetramethylbenzidine solution for 30 min, and finally stopped with 2 M H_2_SO_4_. The absorbance at 450 nm was read by an enzyme counter (Bio-Rad, Hercules, CA). Relative concentrations (ng/mL) were calculated for different antibodies based on their molecular masses compared to VRC01.

For detection of the HIV-1 production from H9-HXB2 after co-culture with CD4-17b-Ab cells, the cell culture supernatants were harvested and measured for the level of viral capsid protein p24 using an HIV-1 p24 ELISA kit (XpressBio, Frederick, MD) according to the manufacturer’s protocol.

### Viral infectivity assay

Pseudoviruses with the Env protein from HIV-1 strain MW965, SF162, X1632, 246F3, CNE55, or CE0217 were produced by co-transfection of 293T cells with the corresponding Env expression plasmid and a pSG3ΔEnv plasmid (NIH-HRP). Viral supernatants were harvested 72 h after transfection and centrifuged at 3,000 rpm for 10 min at 4°C to remove cellular debris. The concentration of the virus was determined using the p24 ELISA kit. The Env-pseudotyped viruses were used at a dose of 20 ng p24 each and incubated with 2 × 10^5^ CD4-17b-Ab cells or UTD cells in 200 µL culture medium for 24 h. Fifty microliters of the supernatants was collected to infect 1 × 10^4^ TZM-bl cells in 96-well plates for 48 h. TZM-bl indicator cells are HeLa-derived cells expressing both CD4 and CCR5 receptors for HIV and are integrated with a Tat-reactive firefly luciferase expression cassette, making them responsive to HIV infection (88). The infected cells were then used to measure the luciferase activity with a substrate of luciferase (Promega, Madison, WI) according to the manufacturer’s protocol.

### Flow cytometry analysis

Cells were analyzed using the LSRForetessa flow cytometer (BD Biosciences). For detection of the expression of cell surface synNotch receptor, transduced cells were incubated with an anti-Myc polyclonal antibody (Proteintech, Rosemont, IL) for 1 h at 4°C, washed three times with PBS, then incubated with a secondary Alexa Fluor Plus 488-goat anti-rabbit IgG (H+L) cross-adsorbed antibody (Invitrogen, Carlsbad, CA) at a 1:500 dilution for 45 min at 4°C, and washed three times with PBS before analysis. To assess Jurkat cell activation, the engineered cells were stained with an anti-CD62L-BV421 antibody and an anti-CD25-APC antibody (BioLegend) for 30 min at 4°C after 24 h of stimulation by target cells. To detect CD8^+^ T cell activation, the engineered cells were stained with a PE-Cy7 or brilliant violet 650 labeled anti-human CD107a antibody (BioLegend) and an APC-Cy7 mouse anti-human CD8 (RPA-T8) antibody (BD Biosciences) for 30 min at 4°C after 24 h of stimulation. For detection of the cell surface CD4 levels, 293T, Jurkat, and U937 cells were stained with an anti-CD4-PE antibody (BD Biosciences) for 30 min at 4°C. All samples were examined at a minimum of 1 × 10^4^ cells per condition. Data were analyzed using the FlowJo software (BD Biosciences). Detailed information on antibodies is provided in Table S1.

### Cytotoxicity assay

To detect paracrine-mediated cytotoxicity, 5 × 10^4^ CD4-17b-Ab Jurkat cells or UTD cells were centrifuged at 400 × *g* for 1 min at a 1:1 ratio with target cells for 20 h. Then, 1 × 10^5^ CD8^+^ T cells were added and co-cultured for 6 h. For CD4-17b-VN CD8^+^ T cell cytotoxicity assays, 1 × 10^5^ CD4-17b-VN CD8^+^ T cells were directly co-incubated with HIV-1_HXB2_ latently infected primary CD4^+^ T cells, either untreated or activated with PMA or JQ1, at a 1:1 ratio for 24 h in 96-well U-bottom plates after centrifugation at 400 × *g* for 1 min. All target cells were pre-labeled with carboxyfluorescein diacetate succinimidyl ester (CFSE; Thermo Fisher Scientific) prior to the addition of receiver cells. The percentage of viable cells in CFSE-positive and 7-AAD (BioLegend)-negative populations was analyzed using flow cytometry before and after the addition of normal or transduced CD8^+^ T cells.

To detect N6-αCD3-mediated specific cytotoxicity against HIV-infected CD4^+^ T cells, an N6-αCD3 expression vector was constructed for protein purification and subsequent detection. The N6-αCD3 gene with a C-terminal histidine tag was cloned into a pVR1012 vector. The expression vector was transferred into Expi293F cells using the protocol provided with an ExpiFectamine 293 Transfection Kit (Thermo Fisher Scientific). The N6-αCD3 protein in the culture supernatant was purified by affinity chromatography, as previously described (44). Subsequently, CD4^+^ T cells were infected with HIV-1_HXB2_ for 2 days. The infected cells were stained using a far-red live/dead staining kit (Invitrogen) for 30 min at 4°C, followed by fixation and permeabilization for 10 min. The cells were then stained with an anti-HIV-1 core antigen-PE antibody (Beckman Coulter, Miami, FL) for 30 min at 4°C. Next, 2 × 10^5^ stained CD4^+^ T cells were co-cultured with CD8^+^ T cells at a 1:1 ratio for 6 h, with or without the addition of 10 ng N6-αCD3 protein. Cell viability and the proportion of p24-positive cells were analyzed by flow cytometry to determine the specific killing of p24-positive cells.

### Statistical analysis

Statistical analysis was carried out using GraphPad Prism (version 7, La Jolla, CA). *P* < 0.05 was considered statistically significant. *, *P* < 0.05; **, *P* < 0.01; ***, *P* < 0.001; and ns, not significant. All data were determined from three or four independent experiments unless otherwise stated. Statistics were calculated using unpaired Student’s *t*-test, Mann-Whitney U-test, or repeated-measures analysis of variance as indicated in each figure legend.

## Data Availability

All relevant data are within the paper and its supplemental material.

## SUPPLEMENTAL MATERIAL

Supplemental material mbio.03839-24-s0001.docx Supplemental text, Fig. S1-S3, and Table S1.

ASM does not own the copyrights to Supplemental Material that may be linked to, or accessed through, an article. The authors have granted ASM a non-exclusive, world-wide license to publish the Supplemental Material files. Please contact the corresponding author directly for reuse.

## References

[1] DeJesus E, Rockstroh JK, Henry K, Molina J-M, Gathe J, Ramanathan S, Wei X, Yale K, Szwarcberg J, White K, Cheng AK, Kearney BP, GS-236-0103 Study Team. 2012. Co-formulated elvitegravir, cobicistat, emtricitabine, and tenofovir disoproxil fumarate versus ritonavir-boosted atazanavir plus co-formulated emtricitabine and tenofovir disoproxil fumarate for initial treatment of HIV-1 infection: a randomised, double-blind, phase 3, non-inferiority trial. Lancet 379:2429–2438. doi:10.1016/S0140-6736(12)60918-0 22748590

[2] Raffi F, Rachlis A, Stellbrink H-J, Hardy WD, Torti C, Orkin C, Bloch M, Podzamczer D, Pokrovsky V, Pulido F, Almond S, Margolis D, Brennan C, Min S, SPRING-2 Study Group. 2013. Once-daily dolutegravir versus raltegravir in antiretroviral-naive adults with HIV-1 infection: 48 week results from the randomised, double-blind, non-inferiority SPRING-2 study. Lancet 381:735–743. doi:10.1016/S0140-6736(12)61853-4 23306000

[3] Sax PE, Wohl D, Yin MT, Post F, DeJesus E, Saag M, Pozniak A, Thompson M, Podzamczer D, Molina JM, Oka S, Koenig E, Trottier B, Andrade-Villanueva J, Crofoot G, Custodio JM, Plummer A, Zhong L, Cao H, Martin H, Callebaut C, Cheng AK, Fordyce MW, McCallister S, GS-US-292-0104/0111 Study Team. 2015. Tenofovir alafenamide versus tenofovir disoproxil fumarate, coformulated with elvitegravir, cobicistat, and emtricitabine, for initial treatment of HIV-1 infection: two randomised, double-blind, phase 3, non-inferiority trials. Lancet 385:2606–2615. doi:10.1016/S0140-6736(15)60616-X 25890673

[4] Eisele E, Siliciano RF. 2012. Redefining the viral reservoirs that prevent HIV-1 eradication. Immunity 37:377–388. doi:10.1016/j.immuni.2012.08.010 22999944 PMC3963158

[5] Arts EJ, Hazuda DJ. 2012. HIV-1 antiretroviral drug therapy. Cold Spring Harb Perspect Med 2:a007161. doi:10.1101/cshperspect.a007161 22474613 PMC3312400

[6] Friis-Møller N, Sabin CA, Weber R, Monforte A, El-Sadr WM, Reiss P, Thiébaut R, Morfeldt L, Wit S, Pradier C, The data collection on adverse events of anti-HIV drugs (DAD) Study Group. 2003. Combination antiretroviral therapy and the risk of myocardial infarction. N Engl J Med 349:1993–2003. doi:10.1056/NEJMoa030218 14627784

[7] Smit C, Geskus R, Walker S, Sabin C, Coutinho R, Porter K, Prins M, CASCADE Collaboration. 2006. Effective therapy has altered the spectrum of cause-specific mortality following HIV seroconversion. AIDS 20:741–749. doi:10.1097/01.aids.0000216375.99560.a2 16514305

[8] Antiretroviral Therapy Cohort Collaboration. 2008. Life expectancy of individuals on combination antiretroviral therapy in high-income countries: a collaborative analysis of 14 cohort studies. Lancet 372:293–299. doi:10.1016/S0140-6736(08)61113-7 18657708 PMC3130543

[9] Fernandez-Montero JV, Eugenia E, Barreiro P, Labarga P, Soriano V. 2013. Antiretroviral drug-related toxicities - clinical spectrum, prevention, and management. Expert Opin Drug Saf 12:697–707. doi:10.1517/14740338.2013.806480 23730950

[10] Deeks SG, Walker BD. 2007. Human immunodeficiency virus controllers: mechanisms of durable virus control in the absence of antiretroviral therapy. Immunity 27:406–416. doi:10.1016/j.immuni.2007.08.010 17892849

[11] Julg B, Pereyra F, Buzón MJ, Piechocka-Trocha A, Clark MJ, Baker BM, Lian J, Miura T, Martinez-Picado J, Addo MM, Walker BD. 2010. Infrequent recovery of HIV from but robust exogenous infection of activated CD4(+) T cells in HIV elite controllers. Clin Infect Dis 51:233–238. doi:10.1086/653677 20550452 PMC3749734

[12] Collins DR, Gaiha GD, Walker BD. 2020. CD8+ T cells in HIV control, cure and prevention. Nat Rev Immunol 20:471–482. doi:10.1038/s41577-020-0274-9 32051540 PMC7222980

[13] Liu B, Zhang W, Xia B, Jing S, Du Y, Zou F, Li R, Lu L, Chen S, Li Y, Hu Q, Lin Y, Zhang Y, He Z, Zhang X, Chen X, Peng T, Tang X, Cai W, Pan T, Li L, Zhang H. 2021. Broadly neutralizing antibody-derived CAR T cells reduce viral reservoir in individuals infected with HIV-1. J Clin Invest 131:e150211. doi:10.1172/JCI150211 34375315 PMC8483761

[14] Pan H, Yang X, Wang J, Liang H, Jiang Z, Zhao L, Wang Y, Liang Z, Shen X, Lin Q, Liang Y, Yang J, Lu P, Zhu Y, Li M, Wang P, Xu J, Lu H, Zhu H. 2023. Allogeneic gene-edited HIV-specific CAR-T cells secreting PD-1 blocking scFv enhance specific cytotoxic activity against HIV Env^+^ cells in vivo. Virol Sin 38:285–295. doi:10.1016/j.virs.2023.01.003 36657565 PMC10176442

[15] Liu B, Zou F, Lu L, Chen C, He D, Zhang X, Tang X, Liu C, Li L, Zhang H. 2016. Chimeric antigen receptor T cells guided by the single-chain Fv of a broadly neutralizing antibody specifically and effectively eradicate virus reactivated from latency in CD4+ T lymphocytes isolated from HIV-1-infected individuals receiving suppressive combined antiretroviral therapy. J Virol 90:9712–9724. doi:10.1128/JVI.00852-16 27535056 PMC5068523

[16] Ali A, Kitchen SG, Chen ISY, Ng HL, Zack JA, Yang OO. 2016. HIV-1-specific chimeric antigen receptors based on broadly neutralizing antibodies. J Virol 90:6999–7006. doi:10.1128/JVI.00805-16 27226366 PMC4944295

[17] Hale M, Mesojednik T, Romano Ibarra GS, Sahni J, Bernard A, Sommer K, Scharenberg AM, Rawlings DJ, Wagner TA. 2017. Engineering HIV-resistant, anti-HIV chimeric antigen receptor T cells. Mol Ther 25:570–579. doi:10.1016/j.ymthe.2016.12.023 28143740 PMC5363191

[18] Anthony-Gonda K, Bardhi A, Ray A, Flerin N, Li M, Chen W, Ochsenbauer C, Kappes JC, Krueger W, Worden A, Schneider D, Zhu Z, Orentas R, Dimitrov DS, Goldstein H, Dropulić B. 2019. Multispecific anti-HIV duoCAR-T cells display broad in vitro antiviral activity and potent in vivo elimination of HIV-infected cells in a humanized mouse model. Sci Transl Med 11:eaav5685. doi:10.1126/scitranslmed.aav5685 31391322 PMC7136029

[19] Maldini CR, Gayout K, Leibman RS, Dopkin DL, Mills JP, Shan X, Glover JA, Riley JL. 2020. HIV-resistant and HIV-specific CAR-modified CD4(+) T cells mitigate HIV disease progression and confer CD4(+) T cell help in vivo. Mol Ther 28:1585–1599. doi:10.1016/j.ymthe.2020.05.012 32454027 PMC7335752

[20] Rust BJ, Kean LS, Colonna L, Brandenstein KE, Poole NH, Obenza W, Enstrom MR, Maldini CR, Ellis GI, Fennessey CM, Huang ML, Keele BF, Jerome KR, Riley JL, Kiem HP, Peterson CW. 2020. Robust expansion of HIV CAR T cells following antigen boosting in ART-suppressed nonhuman primates. Blood 136:1722–1734. doi:10.1182/blood.2020006372 32614969 PMC7544543

[21] Maldini CR, Claiborne DT, Okawa K, Chen T, Dopkin DL, Shan X, Power KA, Trifonova RT, Krupp K, Phelps M, Vrbanac VD, Tanno S, Bateson T, Leslie GJ, Hoxie JA, Boutwell CL, Riley JL, Allen TM. 2020. Dual CD4-based CAR T cells with distinct costimulatory domains mitigate HIV pathogenesis in vivo. Nat Med 26:1776–1787. doi:10.1038/s41591-020-1039-5 32868878 PMC9422086

[22] Zhen A, Peterson CW, Carrillo MA, Reddy SS, Youn CS, Lam BB, Chang NY, Martin HA, Rick JW, Kim J, Neel NC, Rezek VK, Kamata M, Chen ISY, Zack JA, Kiem HP, Kitchen SG. 2017. Long-term persistence and function of hematopoietic stem cell-derived chimeric antigen receptor T cells in a nonhuman primate model of HIV/AIDS. PLoS Pathog 13:e1006753. doi:10.1371/journal.ppat.1006753 29284044 PMC5746250

[23] Haran KP, Hajduczki A, Pampusch MS, Mwakalundwa G, Vargas-Inchaustegui DA, Rakasz EG, Connick E, Berger EA, Skinner PJ. 2018. Simian immunodeficiency virus (SIV)-specific chimeric antigen receptor-T cells engineered to target B cell follicles and suppress SIV replication. Front Immunol 9:492. doi:10.3389/fimmu.2018.00492 29616024 PMC5869724

[24] Mu W, Carrillo MA, Kitchen SG. 2020. Engineering CAR T cells to target the HIV reservoir. Front Cell Infect Microbiol 10:410. doi:10.3389/fcimb.2020.00410 32903563 PMC7438537

[25] Liu B, Zhang W, Zhang H. 2019. Development of CAR-T cells for long-term eradication and surveillance of HIV-1 reservoir. Curr Opin Virol 38:21–30. doi:10.1016/j.coviro.2019.04.004 31132749

[26] Mitsuyasu RT, Anton PA, Deeks SG, Scadden DT, Connick E, Downs MT, Bakker A, Roberts MR, June CH, Jalali S, Lin AA, Pennathur-Das R, Hege KM. 2000. Prolonged survival and tissue trafficking following adoptive transfer of CD4 ζ gene-modified autologous CD4(+) and CD8(+) T cells in human immunodeficiency virus-infected subjects. Blood 96:785–793. doi:10.1182/blood.V96.3.785 10910888

[27] Walker RE, Bechtel CM, Natarajan V, Baseler M, Hege KM, Metcalf JA, Stevens R, Hazen A, Blaese RM, Chen CC, Leitman SF, Palensky J, Wittes J, Davey RT Jr, Falloon J, Polis MA, Kovacs JA, Broad DF, Levine BL, Roberts MR, Masur H, Lane HC. 2000. Long-term in vivo survival of receptor-modified syngeneic T cells in patients with human immunodeficiency virus infection. Blood 96:467–474. doi:10.1182/blood.V96.2.467 10887107

[28] Deeks SG, Wagner B, Anton PA, Mitsuyasu RT, Scadden DT, Huang C, Macken C, Richman DD, Christopherson C, June CH, Lazar R, Broad DF, Jalali S, Hege KM. 2002. A phase II randomized study of HIV-specific T-cell gene therapy in subjects with undetectable plasma viremia on combination antiretroviral therapy. Mol Ther 5:788–797. doi:10.1006/mthe.2002.0611 12027564

[29] Scholler J, Brady TL, Binder-Scholl G, Hwang WT, Plesa G, Hege KM, Vogel AN, Kalos M, Riley JL, Deeks SG, Mitsuyasu RT, Bernstein WB, Aronson NE, Levine BL, Bushman FD, June CH. 2012. Decade-long safety and function of retroviral-modified chimeric antigen receptor T cells. Sci Transl Med 4:132ra53. doi:10.1126/scitranslmed.3003761 PMC4368443 22553251

[30] Yang H, Wallace Z, Dorrell L. 2018. Therapeutic targeting of HIV reservoirs: how to give T cells a new direction. Front Immunol 9:2861. doi:10.3389/fimmu.2018.02861 30564246 PMC6288286

[31] Julg B, Pegu A, Abbink P, Liu J, Brinkman A, Molloy K, Mojta S, Chandrashekar A, Callow K, Wang K, Chen X, Schmidt SD, Huang J, Koup RA, Seaman MS, Keele BF, Mascola JR, Connors M, Barouch DH. 2017. Virological control by the CD4-binding site antibody N6 in simian-human immunodeficiency virus-infected rhesus monkeys. J Virol 91:e00498-17. doi:10.1128/JVI.00498-17 28539448 PMC5533891

[32] Asokan M, Dias J, Liu C, Maximova A, Ernste K, Pegu A, McKee K, Shi W, Chen X, Almasri C. 2020. Fc-mediated effector function contributes to the in vivo antiviral effect of an HIV neutralizing antibody. Proc Natl Acad Sci U S A 117:18754–18763. doi:10.1073/pnas.2008236117 32690707 PMC7414046

[33] van der Velden YU, Villaudy J, Siteur-van Rijnstra E, van der Linden CA, Vink MA, Schermer EE, Weijer K, Berkhout B, Sanders RW, van Gils MJ. 2020. Diverse HIV-1 escape pathways from broadly neutralizing antibody PGDM1400 in humanized mice. MAbs 12:1845908. doi:10.1080/19420862.2020.1845908 33218286 PMC7755169

[34] Wang P, Gajjar MR, Yu J, Padte NN, Gettie A, Blanchard JL, Russell-Lodrigue K, Liao LE, Perelson AS, Huang Y, Ho DD. 2020. Quantifying the contribution of Fc-mediated effector functions to the antiviral activity of anti-HIV-1 IgG1 antibodies in vivo. Proc Natl Acad Sci U S A 117:18002–18009. doi:10.1073/pnas.2008190117 32665438 PMC7395461

[35] Lynch RM, Boritz E, Coates EE, DeZure A, Madden P, Costner P, Enama ME, Plummer S, Holman L, Hendel CS. 2015. Virologic effects of broadly neutralizing antibody VRC01 administration during chronic HIV-1 infection. Sci Transl Med 7:319ra206. doi:10.1126/scitranslmed.aad5752 PMC12366723 26702094

[36] Caskey M, Klein F, Lorenzi JCC, Seaman MS, West AP, Buckley N, Kremer G, Nogueira L, Braunschweig M, Scheid JF, Horwitz JA, Shimeliovich I, Ben-Avraham S, Witmer-Pack M, Platten M, Lehmann C, Burke LA, Hawthorne T, Gorelick RJ, Walker BD, Keler T, Gulick RM, Fätkenheuer G, Schlesinger SJ, Nussenzweig MC. 2015. Viraemia suppressed in HIV-1-infected humans by broadly neutralizing antibody 3BNC117. Nature 522:487–491. doi:10.1038/nature14411 25855300 PMC4890714

[37] Caskey M, Schoofs T, Gruell H, Settler A, Karagounis T, Kreider EF, Murrell B, Pfeifer N, Nogueira L, Oliveira TY. 2017. Antibody 10-1074 suppresses viremia in HIV-1-infected individuals. Nat Med 23:185–191. doi:10.1038/nm.4268 28092665 PMC5467219

[38] Stephenson KE, Julg B, Tan CS, Zash R, Walsh SR, Rolle C-P, Monczor AN, Lupo S, Gelderblom HC, Ansel JL. 2021. Safety, pharmacokinetics and antiviral activity of PGT121, a broadly neutralizing monoclonal antibody against HIV-1: a randomized, placebo-controlled, phase 1 clinical trial. Nat Med 27:1718–1724. doi:10.1038/s41591-021-01509-0 34621054 PMC8516645

[39] Gaebler C, Nogueira L, Stoffel E, Oliveira TY, Breton G, Millard KG, Turroja M, Butler A, Ramos V, Seaman MS, Reeves JD, Petroupoulos CJ, Shimeliovich I, Gazumyan A, Jiang CS, Jilg N, Scheid JF, Gandhi R, Walker BD, Sneller MC, Fauci A, Chun T-W, Caskey M, Nussenzweig MC. 2022. Prolonged viral suppression with anti-HIV-1 antibody therapy. Nature 606:368–374. doi:10.1038/s41586-022-04597-1 35418681 PMC9177424

[40] Klein F, Mouquet H, Dosenovic P, Scheid JF, Scharf L, Nussenzweig MC. 2013. Antibodies in HIV-1 vaccine development and therapy. Science 341:1199–1204. doi:10.1126/science.1241144 24031012 PMC3970325

[41] Deeks SG, Autran B, Berkhout B, Benkirane M, Cairns S, Chomont N, Chun T-W, Churchill M, Di Mascio M, Katlama C. 2012. Towards an HIV cure: a global scientific strategy. Nat Rev Immunol 12:607–614. doi:10.1038/nri3262 22814509 PMC3595991

[42] Sloan DD, Lam CYK, Irrinki A, Liu L, Tsai A, Pace CS, Kaur J, Murry JP, Balakrishnan M, Moore PA, Johnson S, Nordstrom JL, Cihlar T, Koenig S. 2015. Targeting HIV reservoir in infected CD4 T cells by dual-affinity re-targeting molecules (DARTs) that bind HIV envelope and recruit cytotoxic T cells. PLoS Pathog 11:e1005233. doi:10.1371/journal.ppat.1005233 26539983 PMC4634948

[43] Sung JAM, Pickeral J, Liu L, Stanfield-Oakley SA, Lam C-Y, Garrido C, Pollara J, LaBranche C, Bonsignori M, Moody MA. 2015. Dual-affinity re-targeting proteins direct T cell-mediated cytolysis of latently HIV-infected cells. J Clin Invest 125:4077–4090. doi:10.1172/JCI82314 26413868 PMC4639974

[44] Brozy J, Schlaepfer E, Mueller CKS, Rochat MA, Rampini SK, Myburgh R, Raum T, Kufer P, Baeuerle PA, Muenz M, Speck RF. 2018. Antiviral activity of HIV gp120-targeting bispecific T cell engager antibody constructs. J Virol 92:e00491-18. doi:10.1128/JVI.00491-18 29720517 PMC6026749

[45] Goebeler M-E, Bargou RC. 2020. T cell-engaging therapies - BiTEs and beyond. Nat Rev Clin Oncol 17:418–434. doi:10.1038/s41571-020-0347-5 32242094

[46] Schriek AI, Aldon YLT, van Gils MJ, de Taeye SW. 2024. Next-generation bNAbs for HIV-1 cure strategies. Antiviral Res 222:105788. doi:10.1016/j.antiviral.2023.105788 38158130

[47] Teixeira AP, Fussenegger M. 2024. Synthetic gene circuits for regulation of next-generation cell-based therapeutics. Adv Sci (Weinh) 11:e2309088. doi:10.1002/advs.202309088 38126677 PMC10885662

[48] Morsut L, Roybal KT, Xiong X, Gordley RM, Coyle SM, Thomson M, Lim WA. 2016. Engineering customized cell sensing and response behaviors using synthetic notch receptors. Cell 164:780–791. doi:10.1016/j.cell.2016.01.012 26830878 PMC4752866

[49] Roybal KT, Williams JZ, Morsut L, Rupp LJ, Kolinko I, Choe JH, Walker WJ, McNally KA, Lim WA. 2016. Engineering T cells with customized therapeutic response programs using synthetic notch receptors. Cell 167:419–432. doi:10.1016/j.cell.2016.09.011 27693353 PMC5072533

[50] Matsuda K, Maeda K. 2024. HIV reservoirs and treatment strategies toward curing HIV infection. Int J Mol Sci 25:2621. doi:10.3390/ijms25052621 38473868 PMC10932120

[51] Allen GM, Frankel NW, Reddy NR, Bhargava HK, Yoshida MA, Stark SR, Purl M, Lee J, Yee JL, Yu W, Li AW, Garcia KC, El-Samad H, Roybal KT, Spitzer MH, Lim WA. 2022. Synthetic cytokine circuits that drive T cells into immune-excluded tumors. Science 378:eaba1624. doi:10.1126/science.aba1624 36520915 PMC9970000

[52] Lagenaur LA, Villarroel VA, Bundoc V, Dey B, Berger EA. 2010. sCD4-17b bifunctional protein: extremely broad and potent neutralization of HIV-1 Env pseudotyped viruses from genetically diverse primary isolates. Retrovirology (Auckl) 7:11. doi:10.1186/1742-4690-7-11 PMC2843639 20158904

[53] Liu L, Patel B, Ghanem MH, Bundoc V, Zheng Z, Morgan RA, Rosenberg SA, Dey B, Berger EA. 2015. Novel CD4-based bispecific chimeric antigen receptor designed for enhanced anti-HIV potency and absence of HIV entry receptor activity. J Virol 89:6685–6694. doi:10.1128/JVI.00474-15 25878112 PMC4468509

[54] Matsunaga S, Jeremiah SS, Miyakawa K, Kurotaki D, Shizukuishi S, Watashi K, Nishitsuji H, Kimura H, Tamura T, Yamamoto N, Shimotohno K, Wakita T, Ryo A. 2020. Engineering cellular biosensors with customizable antiviral responses targeting hepatitis B virus. iScience 23:100867. doi:10.1016/j.isci.2020.100867 32105634 PMC7113479

[55] Huang X, Williams JZ, Chang R, Li Z, Burnett CE, Hernandez-Lopez R, Setiady I, Gai E, Patterson DM, Yu W, Roybal KT, Lim WA, Desai TA. 2021. DNA scaffolds enable efficient and tunable functionalization of biomaterials for immune cell modulation. Nat Nanotechnol 16:214–223. doi:10.1038/s41565-020-00813-z 33318641 PMC7878327

[56] Garibyan M, Hoffman T, Makaske T, Do SK, Wu Y, Williams BA, March AR, Cho N, Pedroncelli N, Lima RE, Soto J, Jackson B, Santoso JW, Khademhosseini A, Thomson M, Li S, McCain ML, Morsut L. 2024. Engineering programmable material-to-cell pathways via synthetic notch receptors to spatially control differentiation in multicellular constructs. Nat Commun 15:5891. doi:10.1038/s41467-024-50126-1 39003263 PMC11246427

[57] Caskey M, Klein F, Nussenzweig MC. 2019. Broadly neutralizing anti-HIV-1 monoclonal antibodies in the clinic. Nat Med 25:547–553. doi:10.1038/s41591-019-0412-8 30936546 PMC7322694

[58] Zhou T, Georgiev I, Wu X, Yang Z-Y, Dai K, Finzi A, Do Kwon Y, Scheid JF, Shi W, Xu L, Yang Y, Zhu J, Nussenzweig MC, Sodroski J, Shapiro L, Nabel GJ, Mascola JR, Kwong PD. 2010. Structural basis for broad and potent neutralization of HIV-1 by antibody VRC01. Science 329:811–817. doi:10.1126/science.1192819 20616231 PMC2981354

[59] Huang J, Kang BH, Ishida E, Zhou T, Griesman T, Sheng Z, Wu F, Doria-Rose NA, Zhang B, McKee K. 2016. Identification of a CD4-binding-site antibody to HIV that evolved near-pan neutralization breadth. Immunity 45:1108–1121. doi:10.1016/j.immuni.2016.10.027 27851912 PMC5770152

[60] Kwon YD, Georgiev IS, Ofek G, Zhang B, Asokan M, Bailer RT, Bao A, Caruso W, Chen X, Choe M. 2016. Optimization of the solubility of HIV-1-neutralizing antibody 10E8 through somatic variation and structure-based design. J Virol 90:5899–5914. doi:10.1128/JVI.03246-15 27053554 PMC4907239

[61] Kwon YD, Chuang G-Y, Zhang B, Bailer RT, Doria-Rose NA, Gindin TS, Lin B, Louder MK, McKee K, O’Dell S. 2018. Surface-matrix screening identifies semi-specific interactions that improve potency of a near pan-reactive HIV-1-neutralizing antibody. Cell Rep 22:1798–1809. doi:10.1016/j.celrep.2018.01.023 29444432 PMC5889116

[62] Xu L, Pegu A, Rao E, Doria-Rose N, Beninga J, McKee K, Lord DM, Wei RR, Deng G, Louder M. 2017. Trispecific broadly neutralizing HIV antibodies mediate potent SHIV protection in macaques. Science 358:85–90. doi:10.1126/science.aan8630 28931639 PMC5978417

[63] Pegu A, Asokan M, Wu L, Wang K, Hataye J, Casazza JP, Guo X, Shi W, Georgiev I, Zhou T, Chen X, O’Dell S, Todd J-P, Kwong PD, Rao SS, Yang Z, Koup RA, Mascola JR, Nabel GJ. 2015. Activation and lysis of human CD4 cells latently infected with HIV-1. Nat Commun 6:8447. doi:10.1038/ncomms9447 26485194 PMC4633990

[64] Klinger M, Benjamin J, Kischel R, Stienen S, Zugmaier G. 2016. Harnessing T cells to fight cancer with BiTE antibody constructs--past developments and future directions. Immunol Rev 270:193–208. doi:10.1111/imr.12393 26864113

[65] Ruffo E, Butchy AA, Tivon Y, So V, Kvorjak M, Parikh A, Adams EL, Miskov-Zivanov N, Finn OJ, Deiters A, Lohmueller J. 2023. Post-translational covalent assembly of CAR and synNotch receptors for programmable antigen targeting. Nat Commun 14:2463. doi:10.1038/s41467-023-37863-5 37160880 PMC10169838

[66] Clouse KA, Powell D, Washington I, Poli G, Strebel K, Farrar W, Barstad P, Kovacs J, Fauci AS, Folks TM. 1989. Monokine regulation of human immunodeficiency virus-1 expression in a chronically infected human T cell clone. J Immunol 142:431–438. 2463307

[67] Ao Z, Zhu R, Tan X, Liu L, Chen L, Liu S, Yao X. 2016. Activation of HIV-1 expression in latently infected CD4+ T cells by the small molecule PKC412. Virol J 13:177. doi:10.1186/s12985-016-0637-9 27769267 PMC5073835

[68] Yang X, Zhao X, Zhu Y, Xun J, Wen Q, Pan H, Yang J, Wang J, Liang Z, Shen X, Liang Y, Lin Q, Liang H, Li M, Chen J, Jiang S, Xu J, Lu H, Zhu H. 2022. FBXO34 promotes latent HIV-1 activation by post-transcriptional modulation. Emerg Microbes Infect 11:2785–2799. doi:10.1080/22221751.2022.2140605 36285453 PMC9665091

[69] Hütter G, Nowak D, Mossner M, Ganepola S, Müssig A, Allers K, Schneider T, Hofmann J, Kücherer C, Blau O, Blau IW, Hofmann WK, Thiel E. 2009. Long-term control of HIV by CCR5 Delta32/Delta32 stem-cell transplantation. N Engl J Med 360:692–698. doi:10.1056/NEJMoa0802905 19213682

[70] Gupta RK, Abdul-Jawad S, McCoy LE, Mok HP, Peppa D, Salgado M, Martinez-Picado J, Nijhuis M, Wensing AMJ, Lee H, Grant P, Nastouli E, Lambert J, Pace M, Salasc F, Monit C, Innes AJ, Muir L, Waters L, Frater J, Lever AML, Edwards SG, Gabriel IH, Olavarria E. 2019. HIV-1 remission following CCR5Δ32/Δ32 haematopoietic stem-cell transplantation. Nature 568:244–248. doi:10.1038/s41586-019-1027-4 30836379 PMC7275870

[71] Gupta RK, Peppa D, Hill AL, Gálvez C, Salgado M, Pace M, McCoy LE, Griffith SA, Thornhill J, Alrubayyi A, Huyveneers LEP, Nastouli E, Grant P, Edwards SG, Innes AJ, Frater J, Nijhuis M, Wensing AMJ, Martinez-Picado J, Olavarria E. 2020. Evidence for HIV-1 cure after CCR5Δ32/Δ32 allogeneic haemopoietic stem-cell transplantation 30 months post analytical treatment interruption: a case report. Lancet HIV 7:e340–e347. doi:10.1016/S2352-3018(20)30069-2 32169158 PMC7606918

[72] Hsu J, Van Besien K, Glesby MJ, Pahwa S, Coletti A, Warshaw MG, Petz L, Moore TB, Chen YH, Pallikkuth S, Dhummakupt A, Cortado R, Golner A, Bone F, Baldo M, Riches M, Mellors JW, Tobin NH, Browning R, Persaud D, Bryson Y, International maternal pediatric adolescent AIDS clinical trials network (IMPAACT) P1107 team. 2023. HIV-1 remission and possible cure in a woman after haplo-cord blood transplant. Cell 186:1115–1126. doi:10.1016/j.cell.2023.02.030 36931242 PMC10616809

[73] Powell AB, Ren Y, Korom M, Saunders D, Hanley PJ, Goldstein H, Nixon DF, Bollard CM, Lynch RM, Jones RB, Cruz CRY. 2020. Engineered antigen-specific T cells secreting broadly neutralizing antibodies: combining innate and adaptive immune response against HIV. Mol Ther Methods Clin Dev 19:78–88. doi:10.1016/j.omtm.2020.08.015 33005704 PMC7508916

[74] Mao Y, Liao Q, Zhu Y, Bi M, Zou J, Zheng N, Zhu L, Zhao C, Liu Q, Liu L, Chen J, Gu L, Liu Z, Pan X, Xue Y, Feng M, Ying T, Zhou P, Wu Z, Xiao J, Zhang R, Leng J, Sun Y, Zhang X, Xu J. 2024. Efficacy and safety of novel multifunctional M10 CAR-T cells in HIV-1-infected patients: a phase I, multicenter, single-arm, open-label study. Cell Discov 10:49. doi:10.1038/s41421-024-00658-z 38740803 PMC11091177

[75] Bargou R, Leo E, Zugmaier G, Klinger M, Goebeler M, Knop S, Noppeney R, Viardot A, Hess G, Schuler M, Einsele H, Brandl C, Wolf A, Kirchinger P, Klappers P, Schmidt M, Riethmüller G, Reinhardt C, Baeuerle PA, Kufer P. 2008. Tumor regression in cancer patients by very low doses of a T cell-engaging antibody. Science 321:974–977. doi:10.1126/science.1158545 18703743

[76] Moore PA, Zhang W, Rainey GJ, Burke S, Li H, Huang L, Gorlatov S, Veri MC, Aggarwal S, Yang Y, Shah K, Jin L, Zhang S, He L, Zhang T, Ciccarone V, Koenig S, Bonvini E, Johnson S. 2011. Application of dual affinity retargeting molecules to achieve optimal redirected T-cell killing of B-cell lymphoma. Blood 117:4542–4551. doi:10.1182/blood-2010-09-306449 21300981

[77] Promsote W, Xu L, Hataye J, Fabozzi G, March K, Almasri CG, DeMouth ME, Lovelace SE, Talana CA, Doria-Rose NA. 2023. Trispecific antibody targeting HIV-1 and T cells activates and eliminates latently-infected cells in HIV/SHIV infections. Nat Commun 14:3719. doi:10.1038/s41467-023-39265-z 37349337 PMC10287722

[78] Bruel T, Guivel-Benhassine F, Amraoui S, Malbec M, Richard L, Bourdic K, Donahue DA, Lorin V, Casartelli N, Noël N, Lambotte O, Mouquet H, Schwartz O. 2016. Elimination of HIV-1-infected cells by broadly neutralizing antibodies. Nat Commun 7:10844. doi:10.1038/ncomms10844 26936020 PMC4782064

[79] Malbec M, Porrot F, Rua R, Horwitz J, Klein F, Halper-Stromberg A, Scheid JF, Eden C, Mouquet H, Nussenzweig MC, Schwartz O. 2013. Broadly neutralizing antibodies that inhibit HIV-1 cell to cell transmission. J Exp Med 210:2813–2821. doi:10.1084/jem.20131244 24277152 PMC3865481

[80] Ko S-Y, Pegu A, Rudicell RS, Yang Z, Joyce MG, Chen X, Wang K, Bao S, Kraemer TD, Rath T, Zeng M, Schmidt SD, Todd J-P, Penzak SR, Saunders KO, Nason MC, Haase AT, Rao SS, Blumberg RS, Mascola JR, Nabel GJ. 2014. Enhanced neonatal Fc receptor function improves protection against primate SHIV infection. Nature 514:642–645. doi:10.1038/nature13612 25119033 PMC4433741

[81] Edwards JM, Heydarchi B, Khoury G, Salazar-Quiroz NA, Gonelli CA, Wines B, Hogarth PM, Kristensen AB, Parsons MS, Purcell DFJ. 2021. Enhancement of antibody-dependent cellular cytotoxicity and phagocytosis in anti-HIV-1 human-bovine chimeric broadly neutralizing antibodies. J Virol 95:e00219-21. doi:10.1128/JVI.00219-21 33853957 PMC8316091

[82] Kruglova N, Shepelev M. 2024. Increasing gene editing efficiency via CRISPR/Cas9- or Cas12a-mediated Knock-In in primary human T cells. Biomedicines 12:119. doi:10.3390/biomedicines12010119 38255224 PMC10813735

[83] Hyrenius-Wittsten A, Su Y, Park M, Garcia JM, Alavi J, Perry N, Montgomery G, Liu B, Roybal KT. 2021. SynNotch CAR circuits enhance solid tumor recognition and promote persistent antitumor activity in mouse models. Sci Transl Med 13:eabd8836. doi:10.1126/scitranslmed.abd8836 33910981 PMC8594452

[84] Choe JH, Watchmaker PB, Simic MS, Gilbert RD, Li AW, Krasnow NA, Downey KM, Yu W, Carrera DA, Celli A, Cho J, Briones JD, Duecker JM, Goretsky YE, Dannenfelser R, Cardarelli L, Troyanskaya O, Sidhu SS, Roybal KT, Okada H, Lim WA. 2021. SynNotch-CAR T cells overcome challenges of specificity, heterogeneity, and persistence in treating glioblastoma. Sci Transl Med 13:eabe7378. doi:10.1126/scitranslmed.abe7378 33910979 PMC8362330

[85] Zhu I, Liu R, Garcia JM, Hyrenius-Wittsten A, Piraner DI, Alavi J, Israni DV, Liu B, Khalil AS, Roybal KT. 2022. Modular design of synthetic receptors for programmed gene regulation in cell therapies. Cell 185:1431–1443. doi:10.1016/j.cell.2022.03.023 35427499 PMC9108009

[86] Kim Y, Anderson JL, Lewin SR. 2018. Getting the “kill” into “shock and kill”: strategies to eliminate latent HIV. Cell Host Microbe 23:14–26. doi:10.1016/j.chom.2017.12.004 29324227 PMC5990418

[87] Sloas DC, Tran JC, Marzilli AM, Ngo JT. 2023. Tension-tuned receptors for synthetic mechanotransduction and intercellular force detection. Nat Biotechnol 41:1287–1295. doi:10.1038/s41587-022-01638-y 36646932 PMC10499187

[88] Wei X, Decker JM, Liu H, Zhang Z, Arani RB, Kilby JM, Saag MS, Wu X, Shaw GM, Kappes JC. 2002. Emergence of resistant human immunodeficiency virus type 1 in patients receiving fusion inhibitor (T-20) monotherapy. Antimicrob Agents Chemother 46:1896–1905. doi:10.1128/AAC.46.6.1896-1905.2002 12019106 PMC127242

