# Supplementary material for: Engineering of CD8+ T cells with an HIV-specific synthetic notch receptor to secrete broadly therapeutic antibodies for combining antiviral humoral and cellular immune responses
Source: mBio. 2025 Feb 25;16(4):e03839-24. doi: 10.1128/mbio.03839-24 (PMC11980546; doi:10.1128/mbio.03839-24)
Supplement: Supplemental material — Supplemental text, Fig. S1-S3, and Table S1. [file mbio.03839-24-s0001.docx]

**Supplemental Material**

**Supplemental Materials and Methods**

**Analysis of cell surface Env expression**

293T-gp160 cells were stained using an anti-Env 3B3 monoclonal antibody (mAb, NIH-HRP), followed by incubation with a PE-conjugated immunofluorescent secondary antibody (Invitrogen) and nuclear staining with DAPI (4’, 6-diamidino-2-phenylindole) (Beyotime). The stained cells were then examined under a laser scanning confocal microscope (Zeiss, Oberkochen, Germany).

**Western blotting**

Cells were lysed in radio immunoprecipitation assay lysis buffer, incubated on ice for 30 min, and then incubated at 97°C for 10 min to fully denature the proteins. Protein supernatants were collected after the cell lysates were centrifuged at 12,000 rpm for 10 min. The protein samples were separated by SDS-polyacrylamide gel electrophoresis and transferred onto nitrocellulose membranes. The membranes were blocked with 5% nonfat milk and then probed with primary and secondary antibodies. The anti-Env 3B3 mAb and an anti-GAPDH mAb purchased from Proteintech (Rosemont, IL, USA) were used as the primary antibodies. The secondary antibody was a horseradish peroxidase-conjugated anti-mouse IgG secondary antibody (Jackson ImmunoResearch). The protein band intensities were determined using the Adobe Photoshop CC 2019 software (San Jose, CA).

**Supplementary Figure 1**


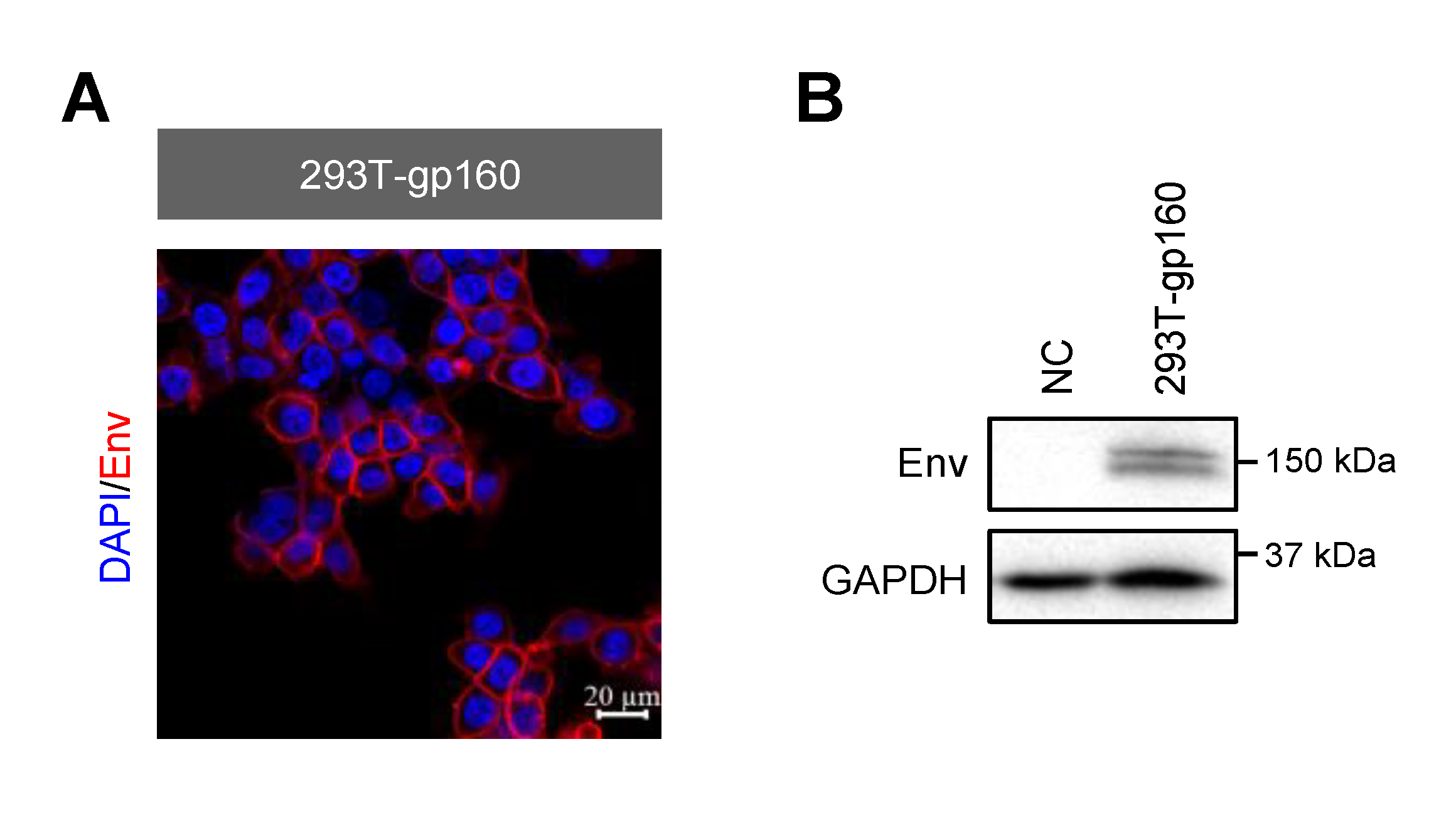


**Figure S1. Characterization of Env expression in the target cells 293T-gp160.**

**(A)** Immunofluorescence detection of viral Env protein (red) expressed on the surface of 293T-gp160 cells. The nucleus was stained with DAPI (blue). The scale bar is 20 μm. **(B)** The expression level of Env in the 293T-gp160 cells was detected by Western blotting. NC, blank 293T cells as the negative control.

**Supplementary Figure 2**


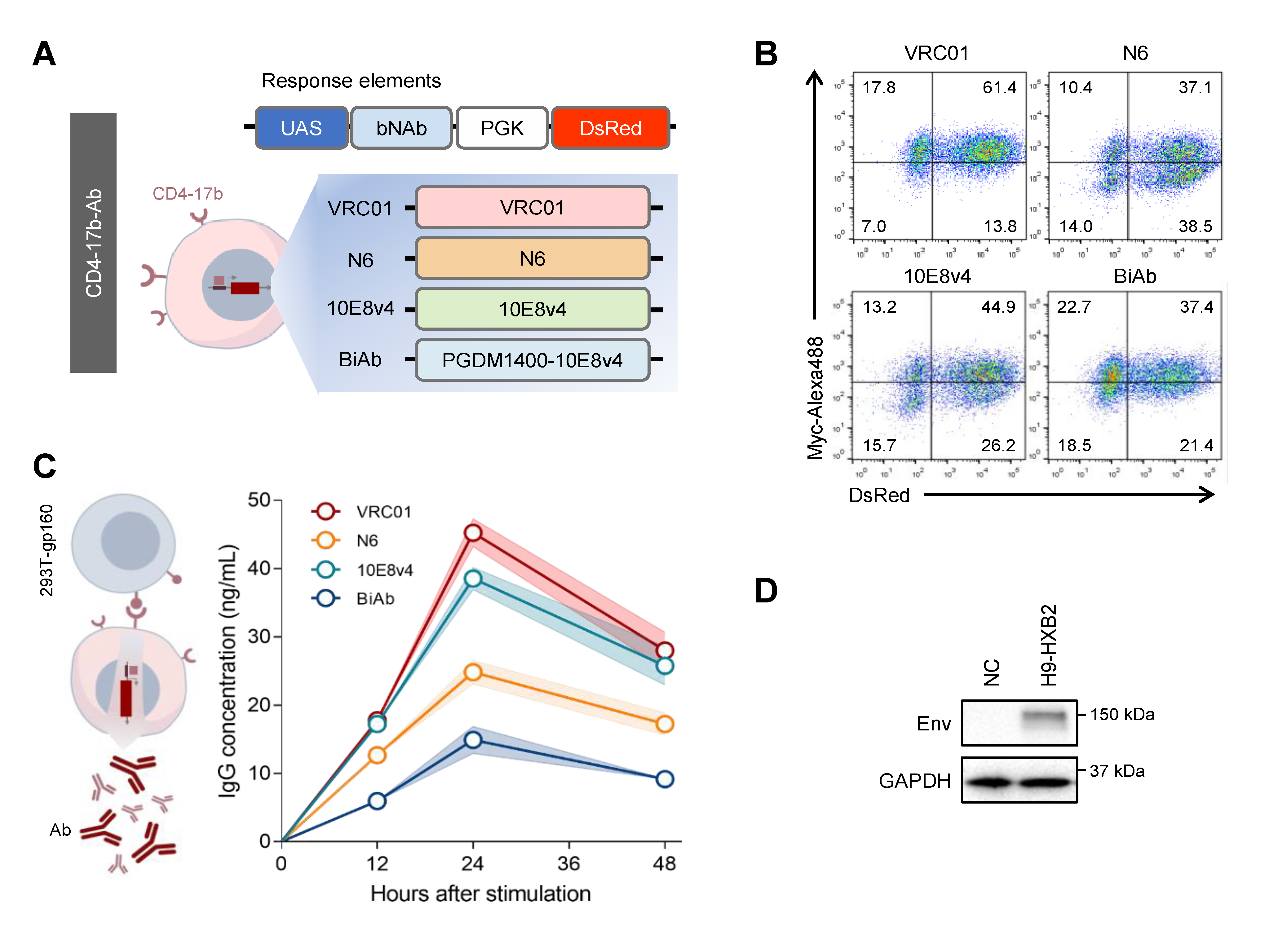


**Figure S2. Design and characterization of the response antibodies used in CD4-17b-Ab cells.**

**(A)** Schematic design of the response bNAbs of the CD4-17b-Ab synNotch circuits. **(B)** The dual-positive rate of CD4-17b-Ab 293T cells analyzed by detecting both Myc-tagged CD4-17b SNR and DsRed using flow cytometry. 293T cells were engineered with CD4-17b SNR and the response element encoding for VRC01, N6, 10E8v4, or BiAb. **(C)** Time course kinetics of IgG secretion of the CD4-17b-Ab cells stimulated with 293T-gp160 cells. The experiments were performed in triplicates and the error bars depict the standard deviation (SD). **(D)** Env expression in HIV-1_HXB2_-infected H9 cells on the third day after infection detected by Western blotting. NC, blank H9 cells.

**Supplementary Figure 3**


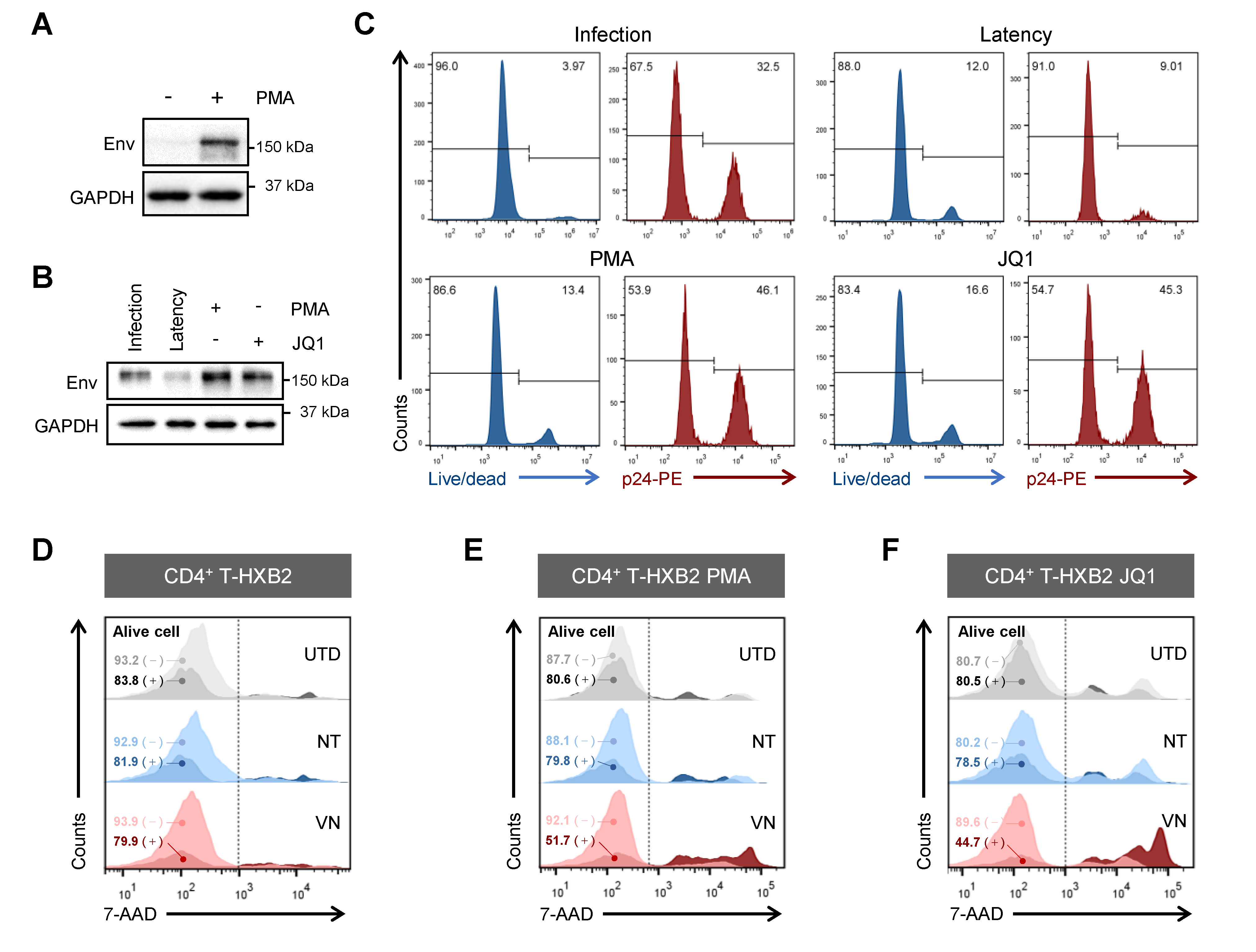


**Figure S3. Characterization of the activation of latently-infected cells and the killing effect of engineered CD8^+^ T cells on the latency-reactivated cells.**

**(A)** Env expression in ACH-2 cells was detected by Western blotting before and after 24 h of PMA treatment. **(B, C)** Env expression detected by Western blotting (B) and p24 expression and cell viability detected by flow cytometry (C) were shown for the primary CD4^+^ T cells after HIV-1_HXB2_ infection, during viral latency, and following reactivation by PMA or JQ1 treatment for 24 h. **(D to F)** The survival of HIV-1_HXB2_ latently-infected CD4^+^ T cells, without (D) or with 24 h of PMA (E) or JQ1 (F) pre-treatment, was analyzed by flow cytometry before and after 24 h of incubation with engineered or UTD CD8^+^ T cells. Anti-CD19 SNR-transduced cells severed as a non-targeting (NT) control. Representative plots from four independent flow cytometry assays are shown in D, E, and F for Figs. 4F, 4G, and 4H, respectively. Light and dark histograms indicate target cell viability before (−) and after (+) the addition of engineered or UTD CD8^+^ T cells.

**Supplementary Table 1**

**Table S1. Information of reagents and resource in this paper.**

| **REAGENT or RESOURCE** | **SOURCE** | **IDENTIFIER** |
| --- | --- | --- |
| **Antibodies** |  |  |
| Myc-tag polyclonal antibody | Proteintect | Cat#16286-1-AP;  RRID: AB_11182162 |
| Alexa Fluor Plus 488-Goat anti-Rabbit IgG | Invitrogen | Cat#ARP-1124 |
| 3B3 monoclonal antibody | NIH HIV reagent program | Cat#ARP-12560 |
| Anti-HIV-1 gp120 monoclonal antibody (VRC01) | NIH HIV reagent program | Cat#12033; |
| 7-AAD viability staining solution | BioLegend | Cat#420403 |
| CellTrace CFSE | Thermo Fisher Scientific | Cat#C34554A |
| Brilliant Violet 650^TM^ anti-human CD107a (LAMP-1) antibody | BioLegend | Cat#328637 |
| Brilliant Violet 421^TM^ anti-human CD62L | BioLegend | Cat#304827;  RRID: AB_2562914 |
| APC anti-human CD25 | BioLegend | Cat#302609;  RRID: AB_314280 |
| APC-Cy^TM^7 mouse anti-human CD8 (RPA-T8) antibody | BD bioscience | Cat#557760;  RRID: AB_396865 |
| anti-CD107a-PE-Cy7 antibody | BD bioscience | Cat#561348;  RRID: AB_10644018 |
| LIVE/DEAD Fixable Far Red Dead Cell Stain Kit | Invitrogen | Cat#L10120 |
| anti-HIV-1 core antigen-PE | Beckman Coulter | Cat#6604667 |
| PE Mouse Anti-human CD4 | BD bioscience | Cat#555347;  RRID: AB_395752 |
| PE-conjugated immunofluorescent secondary antibody | Invitrogen | Cat#31862;RRID: AB_429716 |
| PerCP/Cyanine5.5-labeled F(ab’)2 anti-human IgG | BioLegend | Cat#410709;  RRID: AB_2565787 |
| Ultra-LEAF^TM^ Purified anti-human CD3 | Biolegend | Cat#317347;  RRID: AB_2749889 |
| Ultra-LEAF^TM^ Purified anti-human CD28 | Biolegend | Cat#302943;  RRID: AB_2616667 |
| GAPDH monoclonal antibody | Proteintect | Cat#60004-1-Ig;  RRID: AB_2107436 |
| Goat Anti-Mouse-HRP IgG | Jackson ImmunoResearch | Cat#115-035-003;  RRID: AB_10015289 |
| **Oligonucleotides** | | |
| VRC01-F (Sense: 5’-ACTACGCACGTCCACTTCAG-3’)  VRC01-R (Anti-sense: 5’-CTGATGAGACGATGACCGGG-3’) | This paper | N/A |
| N6-αCD3-F (Sense: 5’-GACACGGGACGTGTACAGAG-3’)  N6-αCD3-R (Anti-sense: 5’-CCCAAGAGGAATCGCCGTAG-3’) | This paper | N/A |
| GAPDH-F (Sense: 5’-AGGTCGGAGTCAACGGAT-3’)  GAPDH-R (Anti-sense: 5’-TCCTGGAAGATGGTGATG-3’) | This paper | N/A |
| **Recombinant DNA** |  |  |
| pHR_PGK_antiCD19_synNotch_Gal4VP64 | Addgene | Cat#79125 |
| pHR_Gal4UAS_tBFP_PGK_mCherry | Addgene | Cat#79130 |
| pHR_PGK_CD4-17b_synNotch_Gal4VP64 | This paper | N/A |
| pHR_Gal4UAS_VRC01_PGK_DsRed | This paper | N/A |
| pHR_Gal4UAS_N6_PGK_DsRed | This paper | N/A |
| pHR_Gal4UAS_10E8v4_PGK_DsRed | This paper | N/A |
| pHR_Gal4UAS_BiAb_PGK_DsRed | This paper | N/A |
| pHR_Gal4UAS_N6-αCD3_PGK_DsRed | This paper | N/A |
| pHR_Gal4UAS_VRC01_P2A_N6-αCD3_PGK_DsRed | This paper | N/A |
| psPAX2 | Addgene | Cat#12260 |
| pMD2.G | Addgene | Cat#12259 |
